# Iron homeostasis, complement, and coagulation cascade as CSF signature of cortical lesions in early multiple sclerosis

**DOI:** 10.1002/acn3.50893

**Published:** 2019-11-01

**Authors:** Roberta Magliozzi, Simon Hametner, Francesco Facchiano, Damiano Marastoni, Stefania Rossi, Marco Castellaro, Alberto Poli, Federico Lattanzi, Andrea Visconti, Richard Nicholas, Richard Reynolds, Salvatore Monaco, Hans Lassmann, Massimiliano Calabrese

**Affiliations:** ^1^ Neurology B Department of Neurosciences, Biomedicine and Movement Sciences University of Verona Verona Italy; ^2^ Division of Brain Sciences Department of Medicine Imperial College London London United Kingdom; ^3^ Neuroimmunology Department Center for Brain Research Medical University of Vienna Wien Austria; ^4^ Department of Oncology and Molecular Oncology Istituto Superiore di Sanità Rome Italy; ^5^ Medical Affairs Department Merck Serono Rome Italy

## Abstract

**Objective:**

Intrathecal inflammation, compartmentalized in cerebrospinal fluid (CSF) and in meningeal infiltrates, has fundamental role in inflammation, demyelination, and neuronal injury in cerebral cortex in multiple sclerosis (MS). Since the exact link between intrathecal inflammation and mechanisms of cortical pathology remains unknown, we aimed to investigate a detailed proteomic CSF profiling which is able to reflect cortical damage in early MS.

**Methods:**

We combined new proteomic method, TRIDENT, CSF analysis, and advanced 3T magnetic resonance imaging (MRI), in 64 MS patients at the time of diagnosis and 26 controls with other neurological disorders. MS patients were stratified according to cortical lesion (CL) load.

**Results:**

We identified 227 proteins differently expressed between the patients with high and low CL load. These were mainly related to complement and coagulation cascade as well as to iron homeostasis pathway (30 and 6% of all identified proteins, respectively). Accordingly, in the CSF of MS patients with high CL load at diagnosis, significantly higher levels of sCD163 (*P* < 0.0001), free hemoglobin (Hb) (*P* < 0.05), haptoglobin (*P* < 0.0001), and fibrinogen (*P* < 0.01) were detected. By contrast, CSF levels of sCD14 were significantly (*P* < 0.05) higher in MS patients with low CL load. Furthermore, CSF levels of sCD163 positively correlated (*P* < 0.01) with CSF levels of neurofilament, fibrinogen, and B cell‐related molecules, such as CXCL13, CXCL12, IL10, and BAFF.

**Interpretation:**

Intrathecal dysregulation of iron homeostasis and coagulation pathway as well as B‐cell and monocyte activity are strictly correlated with cortical damage at early disease stages.

## Introduction

Accumulating neuropathological evidences indicate that multiple sclerosis (MS) is not only characterized by the presence of white matter (WM) demyelinated lesions, readily detectable by conventional magnetic resonance imaging (MRI), but also by focal and diffuse cortical grey matter (GM) demyelination.^1^, ^2^ Advanced MRI methodology has been developed to visualize GM demyelination,^3^, ^4^ which likely plays a key role in the accumulation of motor and cognitive disability in MS, especially during the progressive stages of the disease.

Compartmentalized inflammation within the meninges of MS patients is spatially related to subpial demyelination, which accounts for about 70% of GM demyelination in progressive MS,^5^ and neuronal/glial alterations in the adjacent GM, following a “surface‐in" gradient^6^ from the pial surface toward the WM.

In a recent combined ex vivo and in vivo study, compartmentalized inflammation in meningeal infiltrates and cerebrospinal fluid (CSF) was found significantly associated with increased subpial demyelination.^7^ In particular, a distinctive CSF pro‐inflammatory pattern, including increased levels of CXCL13, TNF, IFN*γ*, CXCL12, IL6, IL8, and IL10, has been associated with a higher GM lesion load both at the time of diagnosis and at death.^7^, ^8^

Several lines of evidence support the notion that regional differences in CSF flow might facilitate focal or diffuse intrathecal trapping of immune cells and stasis of inflammatory molecules, which may diffuse through the pial surface toward the inner cortical layers, in turn mediating a detrimental effect in the adjacent GM.^9^, ^10^

Taken together, these studies suggest a crucial role of CSF/meningeal compartmentalized, intrathecal immune response in the pathogenesis of cortical lesions (CLs). Spurred by the above evidences, using advanced proteomic analysis of CSF combined with advanced MRI techniques, we have attempted to identify more specific molecular pathways involved in early MS‐specific tissue damage at the time of the diagnosis.

## Methods

### Patients

Sixty‐four consecutive treatment‐naïve relapsing‐remitting MS patients from the MS Centre of Verona University Hospital (Italy) were enrolled at diagnosis between March 2015 and February 2016. All the MS patients had a confirmed diagnosis of MS according to Revised McDonald criteria and underwent neurological evaluation, including Expanded Disability Status Scale (EDSS)^11^ assessment, a 3T MRI, and a CSF examination at the time of diagnosis (Table 1). MS patients were stratified in two groups according to the presence of low (n° 28 MSlow, <10 CLs) or high (n° 36 MShigh,>10 CLs) levels of CL number (median value: 10 CLs, range: 0–34) detected using 3D Double Inversion Recovery (DIR) sequences. In addition, we performed CSF analysis in a control group of 26 patients (age, mean, SD:46.2 ± 10.1; 16 female/10 male) with other neurological disorders, including 12 subjects with noninflammatory neurological diseases (NIND) and 14 subjects with other inflammatory neurological diseases (OIND) (Table S1).

**Table 1:** Demographic, clinical, MRI and CSF (protein concentration) details of the two examined MS populations with low and high degree of cortical demyelination.

MS damage	Low (28)	High (36)
Age studied (mean ± SD)	41.6 ± 11.9	39.7 ± 13.8
Gender (f:m)	16:12	23:13
EDSS at recruitment (median; range)	2 (1.0–4.0)	2 (0.0–5.0)
OCBs positive/negative	16/12	34/2
IgG index (mean ± SD)	0.66 ± 0.2	0.92 ± 0.46
CLs volume (mm^3^‐ range)	132 ± 186 (0–575)	1620 ± 728 (365–3910)
Intracortical CLs volume (mm^3^‐ range)	62 ± 109 (0–364)	757 ± 512 (142–2311)
CLs number (mean ± SD)	1.2 ± 1.7 (0–5)	15.7 ± 6.7 (10–34)
Intracortical CLs number (mean ± SD)	0.53 ± 1.03 (0–4)	6.6 ± 4.01 (2–16)
CTh (mm; range)	2.78 ± 0.24 (2.39–3.17)	2.8 ± 0.27 (2.15–3.23)
T2WMLV (cm^3^; range)	6.6 ± 4.2 (2.4–23.3)	5.4 ± 2.2 (1.4–11.7)
Brain volume (cm^3^; mean ± SD)	1513.3 ± 119.7	1506.4 ± 163.7
GM Volume (cm^3^; mean ± SD)	496.8 ± 46.8	494.6 ± 65.3
WM Volume (cm^3^; mean ± SD)	415.1 ± 38.7	402.8 ± 57.6
MIG/CXCL9 (pg/mL; mean ± SD)	24.28 ± 16.21	47.11 ± 45.87
CXCL10/IP10 (pg/mL; mean ± SD)	253.04 ± 264.54	475.84 ± 369.24
SDF1*αβ*/CXCL12 (pg/mL; mean ± SD)	1205.47 ± 842.97	2639.02 ± 1375.13
CXCL13/BCA1 (pg/mL; mean ± SD)	3.11 ± 4.78	29.13 ± 2.968
6Ckine/CCL21 (pg/mL; mean ± SD)	619.08 ± 527.00	1085.29 ± 697.26
GM‐CSF (pg/mL; mean ± SD)	30.40 ± 20.05	59.32 ± 64.78
MIF (pg/mL; mean ± SD)	1534.75 ± 1808.30	2624.57 ± 2805.85
TNF (pg/mL; mean ± SD)	14.23 ± 12.26	43.70 ± 35.40
sTNF‐R1 (pg/mL; mean ± SD)	4357.82 ± 1835.17	11860.49 ± 18986.05
sTNF‐R2 (pg/mL; mean ± SD)	1642.13 ± 902.52	2301.40 ± 1559.97
TWEAK/TNFSF12 (pg/mL; mean ± SD)	1506.11 ± 1158.68	3157.59 ± 2386.68
APRIL/TNFSF13 (pg/mL; mean ± SD)	25564.85 ± 18266.06	59598.08 ± 44038.57
BAFF/TNFSF13B (pg/mL; mean ± SD)	5314.47 ± 2135.23	10001.39 ± 6901.69
LIGHT/TNFSF14 (pg/mL; mean ± SD)	528.62 ± 395.08	1174.72 ± 1001.65
IFN‐G (pg/mL; mean ± SD)	2.51 ± 1.76	8.93 ± 7.63
INF‐alfa 2* (pg/mL; mean ± SD)	16.13 ± 15.86	11.59 ± 8.74
INF‐beta (pg/mL; mean ± SD)	57.37 ± 27.13	68.17 ± 39.66
IFNlambda2* (pg/mL; mean ± SD)	1304.70 ± 1475.29	528.94 ± 655.79
IL‐6 (pg/mL; mean ± SD)	7.50 ± 7.53	11.78 ± 8.07
IL‐8 (pg/mL; mean ± SD)	17.11 ± 8.57	31.66 ± 21.41
IL‐10 (pg/mL; mean ± SD)	4.07 ± 1.67	7.99 ± 3.76
IL‐16 (pg/mL; mean ± SD)	35.11 ± 15.64	64.57 ± 53.41
IL‐12(p40) (pg/mL; mean ± SD)	3.29 ± 6.47	4.24 ± 5.37
IL‐12(p70) (pg/mL; mean ± SD)	0.80 ± 1.00	1.01 ± 1.45
MMP‐2 (pg/mL; mean ± SD)	192.83 ± 111.78	715.47 ± 317.47
Osteopontin (pg/mL; mean ± SD)	114611.26 ± 163433.93	151589.51 ± 157961.25
Pentraxin ‐3 (pg/mL; mean ± SD)	417.62 ± 592.90	818.47 ± 799.79
Chitinase 3 ‐like 1 (pg/mL; mean ± SD)	31930.36 ± 41535.25	59571.12 ± 63352.69
sCD163 (pg/mL; mean ± SD)	35199.65 ± 9514.72	65939.36 ± 13129.70

Abbreviations: EDSS, Expanded Disability Status Scale; OCB, oligoclonal bands; IgG index, immunoglobulin‐G index; CL, cortical lesion; CTh, cortical thickness; T2WMLV, T2 white matter lesion volume; MIG/CXCL9, monokine induced by gamma interferon or chemokine (C‐X‐C motif) ligand 9; CXCL10/IP10, C‐X‐C motif chemokine 10 or interferon gamma‐induced protein 10; SDF1αβ/CXCL12, stromal cell‐derived factor or C‐X‐C motif chemokine 12; CXCL13/BCA, chemokine (C‐X‐C motif) ligand 13 or B lymphocyte chemoattractant; 6Ckine/CCL2, Chemokine (C‐C motif) ligand 21; GM‐CSF, Granulocyte‐macrophage colony‐stimulating factor ; MIF, Macrophage migration inhibitor factor; TNF, tumor necrosis factor; sTNF‐R1, soluble tumour necrosis factor‐receptor 1; sTNF‐R2, soluble tumour necrosis factor‐receptor 2; TWEAK/TNFSF12, TNF‐related weak inducer of apoptosis or tumour necrosis factor ligand superfamily member 12; APRIL/TNFSF13, A proliferation‐inducing ligand, or tumour necrosis factor ligand superfamily member 13; BAFF/TNFSF13B, B cell activating factor or tumour necrosis factor ligand superfamily member 13B; LIGHT/TNFSF14, tumor necrosis factor ligand superfamily member 14 or tumour necrosis factor superfamily member 14; IFN‐G, interferon gamma; IFN‐alfa2, interferon alfa2; IFN‐lambda2, interferon lambda2; IFN‐beta, interferon beta; IL‐6, interleukin 6; IL‐8/CXCL8, interleukin‐8 or (C‐X‐C motif) chemokine ligand 8; IL‐10, interleukin10; IL‐16, interleukin16; IL‐12(p40), interleukin‐12 subunit p40; IL‐12(p70), interleukin‐12 subunit p40; MMP2, matrix metallopeptidase 2; sCD163, soluble‐CD163 (cluster of differentiation 163).

### MRI acquisition protocol and analysis

In each MS patient, MRI was performed at least 2 months after the last relapse using a Philips Achieva 3T MRI Scanner and the following image sets were acquired: (1) 3D T1‐weighted Turbo Field Echo (TFE); (2) 3D Double Inversion Recovery (DIR); (3) 3D Fluid‐Attenuated Inversion Recovery (FLAIR). Optimized parameters of each sequence were set as previously reported.^7^ WM and GM lesion number and load and estimation of cortical thickness were assessed by consensus of experienced observers, as previously described.^7^, ^12^ More specifically, details on MRI analysis are reported in Supplementary Materials (Data S1).

### CSF analysis

#### Immunoassay protein analysis

Cerebrospinal fluid samples of MS patients were obtained at least 2 months after the last relapse and within 1 week of the MRI (ethical approval n° 35315), according to Consensus Guidelines for CSF and Blood Biobanking.^13^ The IgG index and the presence/absence of oligoclonal bands (OCB) for each MS patient are reported in Table 1. The levels of 69 inflammatory mediators (Table 1) were assessed using a combination of immune‐assay multiplex techniques (Bio‐Plex X200 System, BioRad, Hercules, CA) as previously optimized^7^, ^8^ and described in Data S1.

The levels of neurofilament light chain (NF‐L) in CSF were measured using Human NF‐light enzyme‐linked immunosorbent assays (ELISA) kit (MyBioSource, San Diego, CA) according to procedures previously optimized.^7^, ^8^

The levels of CSF sCD14 (Quantikine Human sCD14 Immunoassay, R&D Systems), haptoglobin (Quantikine Human Haptoglobin, R&D Systems), free‐hemoglobin (Hb) (Abcam ab157707), and fibrinogen total antigen (#MBS135523, MyBiosource) were measured in duplicate by ELISA assays according to manufacturer's instructions (Table 2).

#### Proteomic analysis

Six CSF samples obtained from three MS patients representative of the MSlow group and three MS patients representative of the MShigh patients, according to the immunoassay (BioPlex) CSF profiling as described above (Table S2), were selected and analyzed using TRIDENT analysis^14^ with denaturation by three different protocols as previously described^14^ and fully explained in Data S1. The obtained protein datasets for each experiment were analyzed by Database for Annotation, Visualization and Integrated Discovery (DAVID, v6.8) software (https://david.ncifcrf.gov), and gene ontology analyses and protein–protein interactions using STRING software as described in detail in Data S1.

### Statistics

Nonparametric Mann–Whitney *U* tests were used to test differences in MRI, EDSS, and proteomic data between MS and control groups or between MSlow and MShigh groups. Pearson correlation coefficients were calculated to analyze the strength of correlation between clinical, MRI, and CSF proteomic data. False discovery rate (FDR), with a significance level of 0.05, was adopted to correct for multiple testing problem. Statistical analysis was performed using GraphPad PRISM‐8‐Software.

## Results

### MS stratification according to cortical lesion load

Upon stratification of MS population according to median value of CL number, the MShigh group (56%, mean CL number = 15.7 ± 6.7; CL volume = 1620 ± 728 mm^3^) was characterized by about 12‐fold higher numbers and volume of CLs compared to MSlow group (44%, mean CL number = 1.2 ± 1.7; CL volume = 132 ± 186 mm^3^) (Table 1). Similar 12‐fold increase was found when type I leukocortical lesions were excluded (Table 1).

No significant differences were found between the two MS groups regarding age (mean MSlow = 41.6 years; mean MShigh = 39.7 years) and disease duration (mean MSlow = 5.6 years; mean MShigh = 5.6 years). As expected, the female/male ratio showed higher number of females in both groups (MSlow = 16:12; MShigh = 23:13). The median EDSS was 2.0 in both MSlow group (range = 1.0–4.0) and MShigh group (range = 0.0–5.0), (Table 1).

No significant differences were found regarding cortical thickness, T2WMLV, brain volume, GM volume, and WM volume between the two MS cohorts (Table 1).

### CSF immune‐assay analysis

By examining the presence and levels of 69 inflammatory mediators in the CSF of all the examined MS patients versus controls, 29 proteins were found significantly (*P* < 0.01) elevated in MS compared to controls (Table 1). When comparing the two subgroups of MS patients, the levels of 27 of the 29 molecules were found significantly higher (*P* < 0.01) in the CSF of MShigh compared to MSlow group, whereas only interferon α 2 (IFN*α*2) and INF*λ*2 were significantly higher in MSlow than MShigh (*P* < 0.01) (asterisks in Table 1).

### CSF proteomic analysis

**Figure 1:**
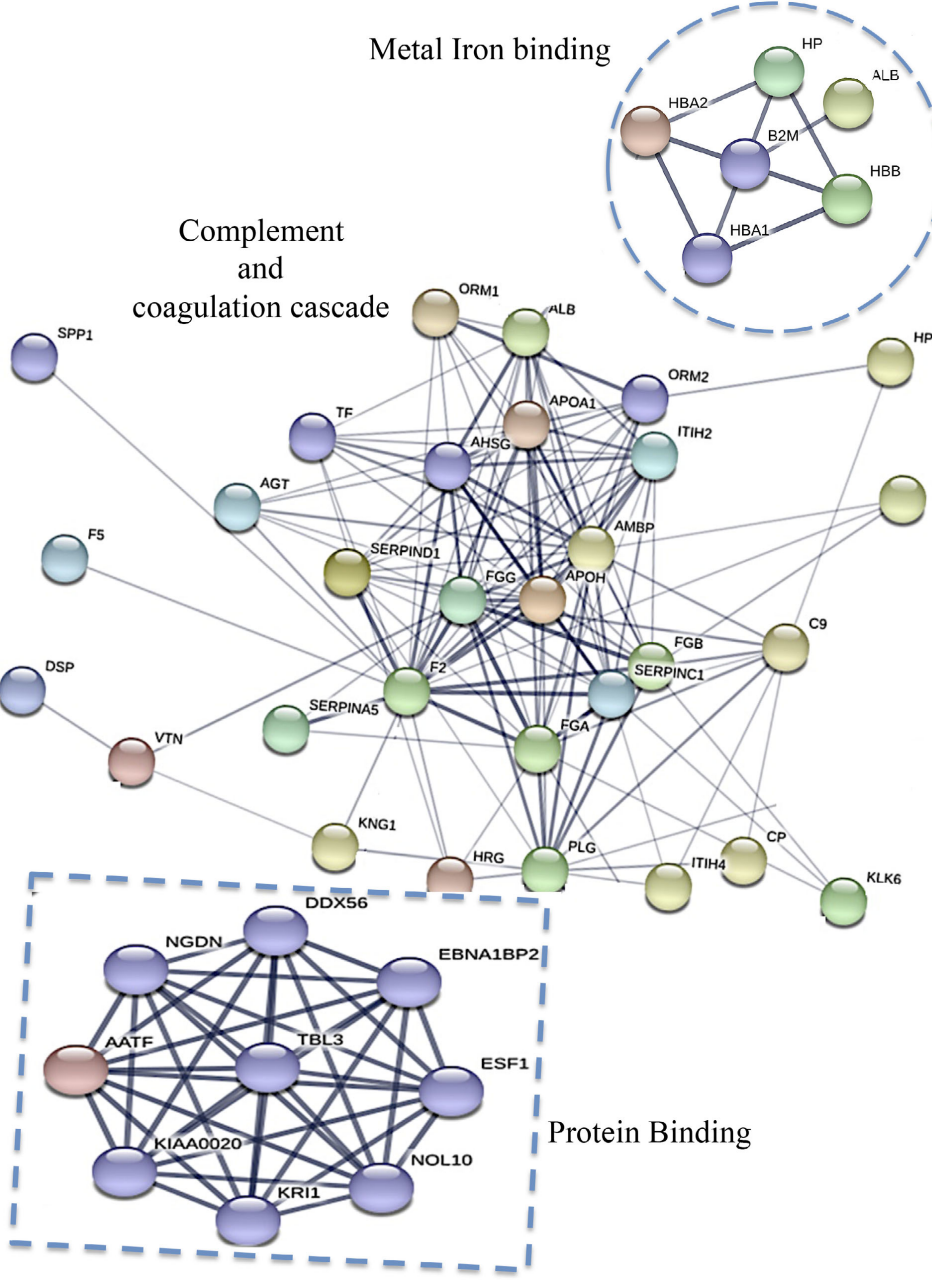
Bioinformatic analysis of proteins identified by proteomic approaches. The protein–protein interaction network was studied and predicted using STRING. The links between proteins represent possible interactions (line thickness indicates the strength of association). The three significant pathways were clustered.

Using TRIDENT approach and mass spectrometry analyses of CSF obtained from three MSlow and three MShigh patients, representative of the two groups identified as above described, a total of 227 proteins were identified to be differently expressed in the CSF of the two examined groups of patients (Table S3). Functional classification analysis (DAVID) identified 10 key pathways differentiating the MShigh and MSlow patients (Table 2; Table S4): complement activation and its regulation (30% of total identified 227 proteins); receptor‐mediated endocytosis (25%); innate immune response (14%); positive regulation of B‐cell activation and B‐cell receptor signaling pathways (7%); cellular iron ion homeostasis (6%); Fc‐gamma receptor signaling pathway involved in phagocytosis (5%); negative regulation of blood coagulation (4%); fibrinolysis (3%); acute phase response (3%); inflammatory response (3%), (Table 2). By applying protein–protein interaction network to further investigate the connection between the 227 total protein identified, 201 nodes and 338 edges were identified in the network. STRING predictive network analysis depicted strong connections among proteins involved in complement and coagulation cascade, cellular iron homeostasis, and protein binding (Fig. 1, Table S4), including, in particular, CD14, Hb, haptoglobin, and fibrinogen.

**Table 2:** List of the 10 main protein pathways differentially expressed in MShigh respect to MSlow patients.

(1) Complement activation, regulation of complement activation (30%)^*^
(2) Receptor‐mediated endocytosis (25%)
(3) Innate immune response (14%)^*^
(4) Positive regulation of B cell activation/B cell receptor signaling pathways (7%)
(5) Cellular iron ion homeostasis (6%)^*^
(6) Fc‐gamma receptor signaling pathway involved in phagocytosis (5%)^*^
(7) Phagocytosis, recognition (4%)^*^
(8) Fibrinolysis (3%)^*^
(9) Acute‐phase response (3%)
(10) Negative regulation of blood coagulation (3%)^*^

The asterisks indicate pathways characterizing by common proteins.

### Differential expression of macrophage CSF biomarkers correlates with cortical lesion load and neurodegeneration

**Figure 2:**
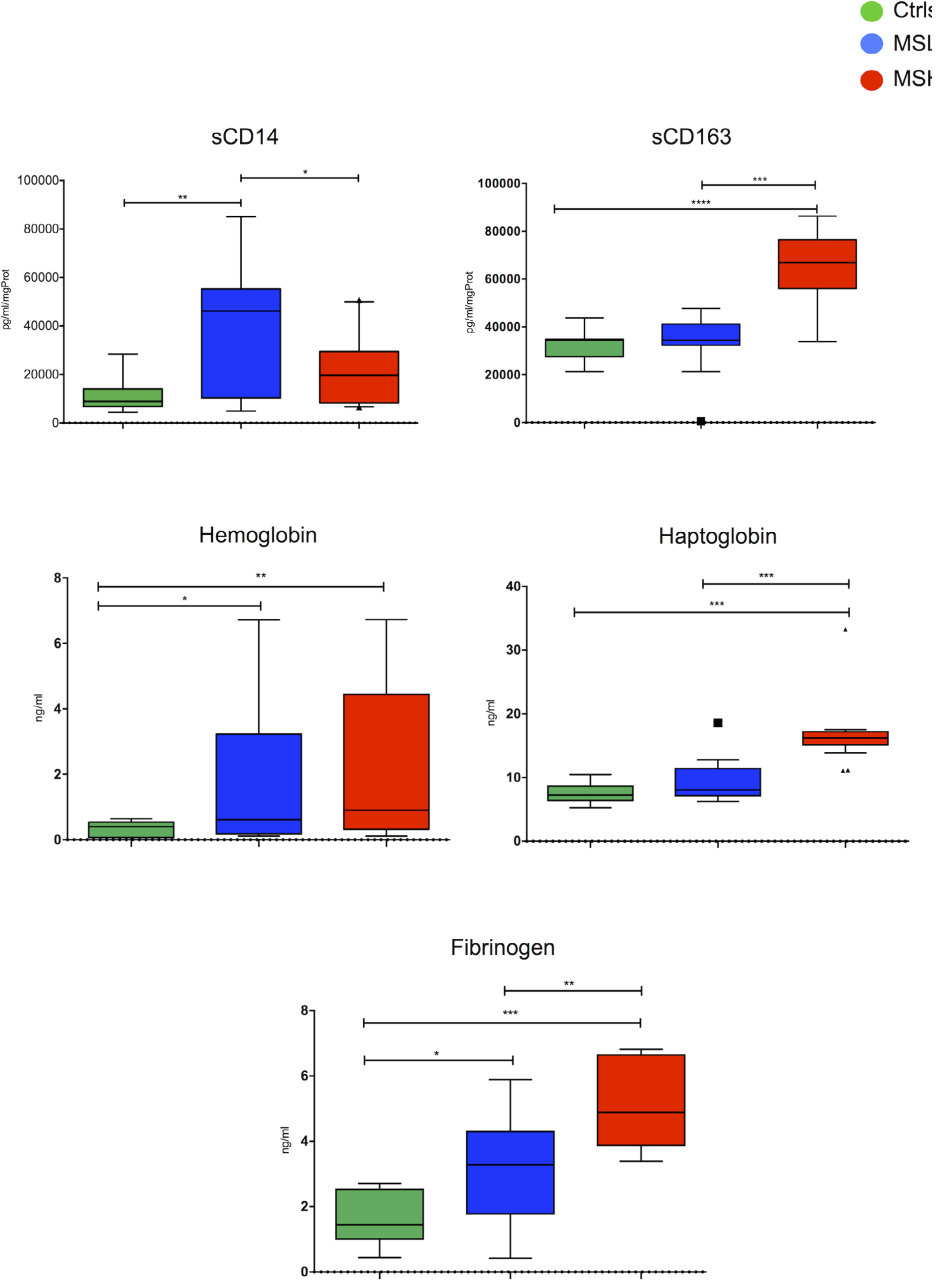
Graphs representing CSF levels of molecules involved in the complement‐coagulation cascade found differentially expressed in MShigh (red bards) respect to MSlow (blue bars) and controls (green bars) and validated by ELISA (sCD14, free‐hemoglobin, haptoglobin, and fibrinogen) or Bio‐Plex methodologies (sCD163). p values for each statistically significant comparison have been reported (*P < 0.05; **P < 0.01; ***P < 0.001).

In order to validate the nonquantitative proteomic analysis on all the 64 examined MS patients and 26 controls, using more quantitative assays, ELISA, we found increased levels of soluble CD14 protein (sCD14) in the CSF of MSlow compared to MShigh patients (*P* < 0.01). Conversely, increased sCD163 was found in the CSF of MShigh in comparison with MSlow patients (*P* < 0.0001; Fig. 2). Free‐Hb levels were significantly higher in the CSF of both MSlow (*P* < 0.05) and MShigh (*P* < 0.01), compared to controls. Haptoglobin levels in the CSF were significantly elevated in the MShigh group (*P* < 0.001), compared to the MSlow and control groups; lastly, CSF fibrinogen levels were significantly higher in MSlow (*P* < 0.05) and MShigh (*P* < 0.001) compared to controls, and in MShigh compared to MSlow (*P* < 0.01) (Fig. 2).

### Differential CSF levels of coagulation cascade and iron homeostasis molecules correlate with cortical lesion load

**Figure 3:**
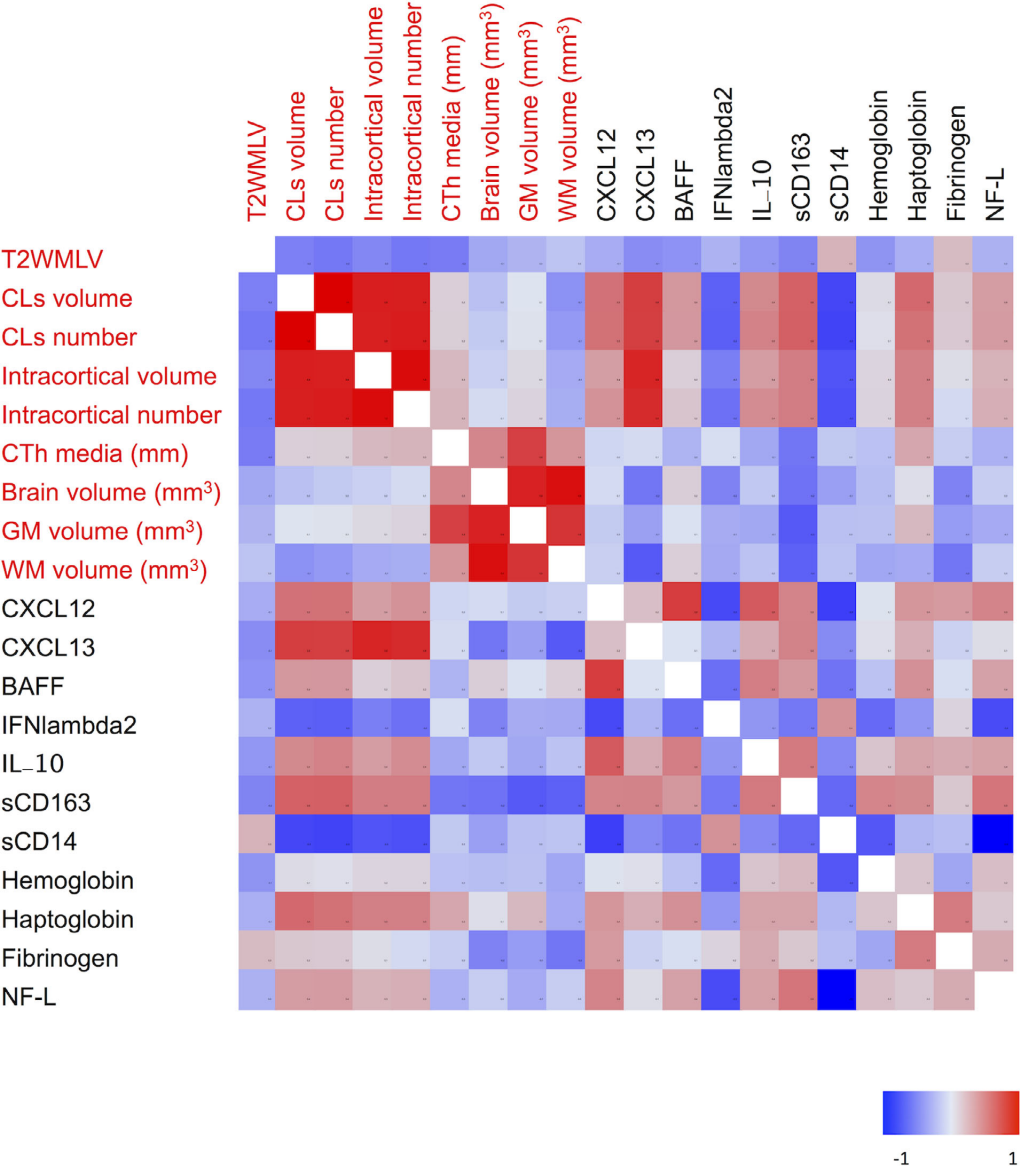
Matrix indicating significant correlations (Pearson correlation) between the examined MRI parameters and CSF biomarkers. Blue color shows negative correlation, red color shows positive correlation; strong colors tonality identifies strongest correlation.

**Figure 4:**
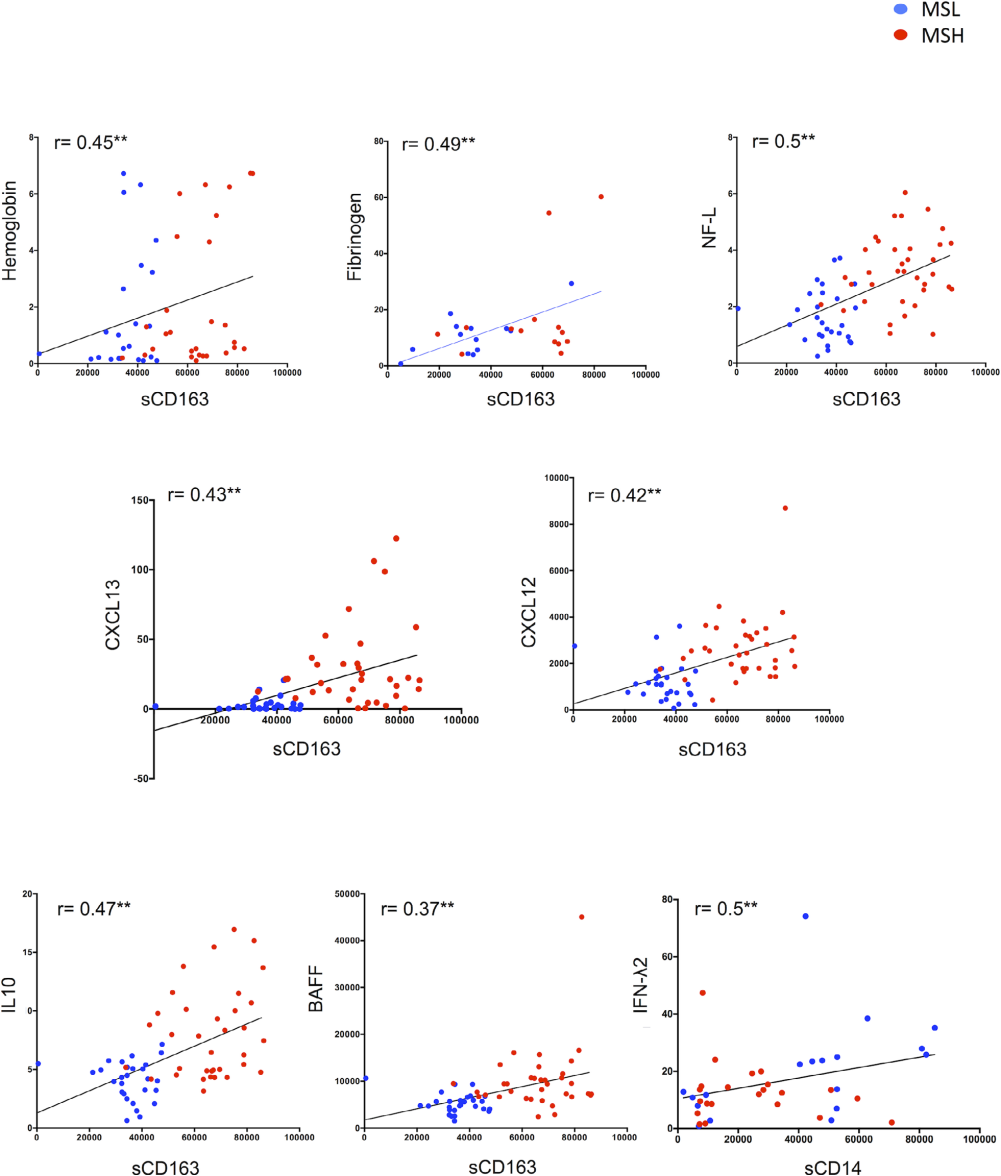
Graphs represent specifically Pearson correlation between CSF levels of sCD163 and hemoglobin, fibrinogen, neurofilament light, CXCL13, CXCL12, IL10, and BAFF. Pearson correlation index and p values for each statistically significant comparison have been reported (*P < 0.05; **P < 0.01).

CSF levels of sCD163 positively correlated with CL volumes (*r* = 0.54; *P* < 0.0001) and numbers (*r* = 0.54; *P* < 0.0001), while an opposite correlation was observed for sCD14 (CL volume: *r* = −0.33; *P* = 0.035; and CL numbers: *r* = −0.34; *P* = 0.030) (Fig. 3). Interestingly, NF‐L correlated positively with sCD163 (*r* = 0.53; *P* < 0.001) and negatively with sCD14 (*r* = −0.51; *P* < 0.001) (Fig. 3). Furthermore, sCD163 correlated positively with several inflammatory mediators of the MShigh CSF profile, including matrix metalloproteinase 2 (MMP2) (*r* = 0.52; *P* < 0.001), IL10 (*r* = 0.47, *P* < 0.001), CXCL13 (*r* = 0.42; *P* < 0.001), and CXCL12 (*r* = 0.42, *P* < 0.001) (Fig. 3). In addition, CSF levels of sCD163 positively correlated with CSF levels of free‐Hb (*r* = 0.44; *P* < 0.001), haptoglobin (*r* = 0.63; *P* < 0.001), and fibrinogen (*r* = 0.492; *P* < 0.01) (Figs. 3 and 4). Interestingly, the two cohorts of examined MS patients were grouped separately, as reflected by the two color clusters in Figure 4.

### B‐cell and macrophage CSF correlations

Cerebrospinal fluid protein levels of sCD163 positively correlated (*P* < 0.001) with the levels of BAFF (*r* = 0.4), IL10 (*r* = 0.5), CXCL13 (*r* = 0.5), CXCL12 (*r* = 0.5), and TNF (*r* = 0.5) (Figs. 3 and 4). In contrast, CSF levels of sCD14 only correlated positively (*r* = 0.50; *P* = 0.0006) with high protein levels of IFN*λ*2 (Figs. 3 and 4).

### Albumin CSF/serum ratio correlates with CSF protein and fibrinogen concentration

**Figure 5:**
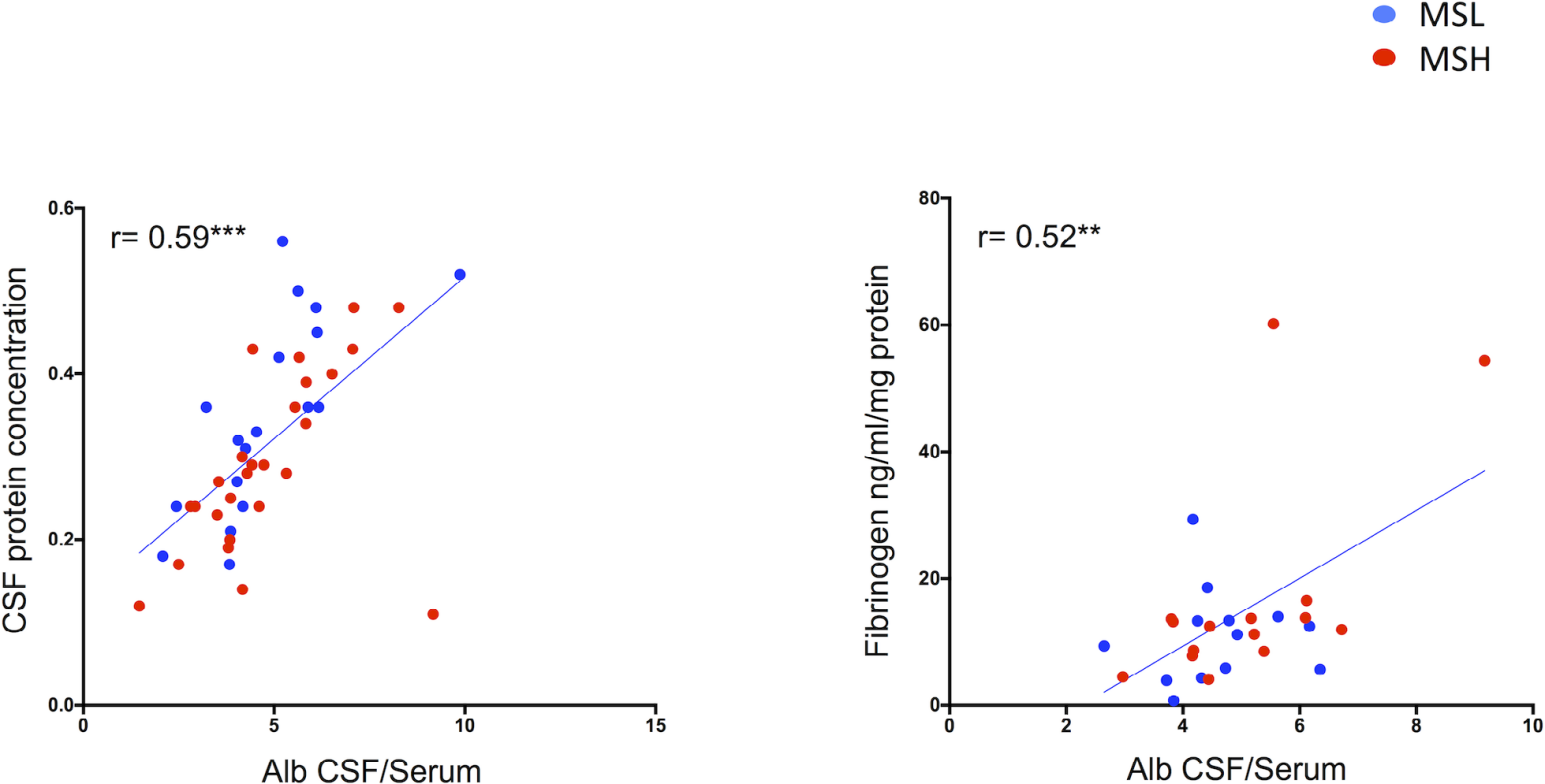
Graphs represent specifically Pearson correlation between albumin CSF/serum ratio and CSF total protein concentration and CSF fibrinogen concentration, respectively. Pearson correlation index and *P* values for each statistically significant comparison have been reported (*P < 0.05; **P < 0.01; ***P < 0.001).

Using CSF/serum albumin ratio as one of the possible indicator of BBB permeability alteration, no significant differences have been detected between MShigh and MSlow patients at the time of diagnosis. Conversely, CSF/serum albumin ratio significantly correlated only with CSF protein concentration (*r* = 0.59; *P* < 0.01) and with CSF fibrinogen levels (*r* = 0.522; *P* < 0.01) (Fig. 5), but not with the other examined biomarkers.

## Discussion

The present study, besides confirming our previous findings of a strong association between severe cortical pathology and a distinctive CSF inflammatory profile^7^ in an independent MS subgroup, enabled us to detect previously unexpected or unknown candidate molecules involved in altered CSF profiles and associated cortical pathology since early disease stages.

In particular, we identified, as strictly associated with severe cortical pathology, a group of molecules involved in the uptake and metabolism of iron, including free‐Hb, haptoglobin, and sCD163. Previous studies have indicated disturbed iron metabolism as a potential amplification factor for demyelination and neurodegeneration in MS patients, particularly in those with progressive disease.^15^ Iron accumulates with aging in human brain mainly in oligodendrocytes and myelin and may be liberated into the extracellular space in the course of MS‐related demyelination, potentially amplifying oxidative injury.^15^ Our current study additionally suggests iron‐containing free‐Hb as an another important pathogenetic factor,we found significantly increased CSF free‐Hb concentrations particularly in MS patients with high CL load. So far, few studies have investigated the role of Hb in MS pathogenesis. In a recent study of a well‐characterized cohort of 140 secondary progressive MS (SPMS) patients,^16^ the rate of brain atrophy significantly correlated with increased serum concentration of alpha‐Hb and beta‐Hb together with enhanced serum lactate dehydrogenase activity in SPMS patients. Our results provide robust evidence for accumulation of free‐Hb in the CSF of MS patients, particularly in those patients with severe cortical pathology. Free‐Hb can be oxidized to methemoglobin (containing ferric iron), ferryl heme intermediate (Fe4+), hemichromes, and free heme or iron, and can cause oxidative injury to lipids, nucleic acids, and proteins.^17^ Uncomplexed heme is instable and either decomposes or is enzymatically oxidized to biliverdin, which is rapidly metabolized to bilirubin, ferrous iron (Fe2+), and carbon monoxide (CO).^18^ Thus, free‐Hb may directly lead to oxidative damage of oligodendrocytes, maltose‐binding protein (MBP), and myelin lipids, and involve in the formation of globin radicals and heme transfer.^19^ Free‐Hb also influences endothelial tight junction proteins, such as zonula occludens‐1 (ZO‐1) and claudin‐5, leading to BBB dysfunction due to loosening of tight junctions,^20^ which may lead to the entry of leukocytes into the cortical parenchyma. For all these reasons, free‐Hb in the CSF of MS patients may be deleterious by increasing oxidative injury in underlying cortex.

Our study has shown a parallel increase in CSF levels of haptoglobin and soluble CD163 (sCD163) molecules involved in transport and macrophage uptake of free‐Hb. Haptoglobin binds Hb with very high affinity, thus representing an efficient buffering mechanism for free‐Hb liberated from damaged erythrocytes.^21^ Binding of Hb to haptoglobin and formation of haptoglobin‐Hb complexes are essential for cellular uptake of Hb via CD163.^21^ CD163 is the haptoglobin‐Hb complex receptor, expressed by monocytes, macrophages, and dendritic cells, which mediates endocytosis of haptoglobin‐Hb complexes in a Ca^2+^‐dependent manner.^22^ The soluble form of CD163 (sCD163), which we detected in the CSF of MS patients with high CL load, may be formed via ectodomain shedding, that is, proteolytic cleavage of the extracellular domain from macrophage membrane‐bound CD163.^23^ Shedding of CD163 may be induced by inflammatory stimuli such as IL10, IL6, glucocorticoids,^24^, ^25^ and by inflammation‐responsive protease ADAM17, which also cleaves pro‐TNF to bioactive TNF.^23^ sCD163 has been proposed as a potential predictor of MS activity^26^ as it was found expressed on macrophages and microglia in MS plaques,^27^ and increased sCD163 levels have been shown in patients with different stages of MS both in serum^28^ and CSF.^26^ As CD163 shedding likely reduces the detoxification capacity of macrophages for free‐Hb from the extracellular space,^23^ we suppose that the presence of sCD163 might be related to reduced Hb detoxification via macrophages and, as a consequence, increased toxic free‐Hb in CSF.

On the contrary, we found elevated CSF levels of sCD14, an innate immune receptor expressed on monocyte, in MS patients with low CL load. The dissimilar sCD14/sCD163 levels detected in the CSF of patients with different degree of GM damage at the time of diagnosis suggest that an altered balance of monocyte profile and activation that may possibly be linked with different amounts of GM injury. To date, discordant data are available on the correlation between blood CD14 expression and disease activity.^25^, ^29^ In particular, an inverse correlation was found between serum levels of sCD14 and disease activity, showing that CD14 may be a good indicator of stable MS.^30^ Thus, a more comprehensive analysis of the CD14/CD163 CSF balance at the time of diagnosis is mandatory as it may indicate a new potential tool to better evaluate the intrathecal stage of macrophage activation to predict and monitor MS evolution.

The correlation found between CSF levels of sCD163 and NF‐L may reflect the activity of CD163^+^ cells in destruction or phagocytosis of neurons and axons.^31^ This finding suggests that combined analysis of CSF sCD163 and NF‐L levels at diagnosis may represent a possible early indicator of CL activity.

In addition, we found a pronounced increase of fibrinogen, normally absent in healthy brain, in the CSF of MS patients at early stage of the disease with high GM lesion load. Fibrinogen deposition was previously found associated with blood brain barrier disruption, neuroinflammation, and neurodegeneration in MS and several other neurological conditions.^32^, ^33^ In addition, fibrinogen was found associated with reduced neuronal density in progressive MS CLs^33^ suggesting that it can have a key role in disability accumulation, in particular, in the progressive phase of the disease. Fibrinogen activates the bone morphogenetic protein (BMP) signaling pathway in OPCs, thus suppressing remyelination.^34^ Fibrinogen was recently detected at the edge of chronic active but not inactive lesions of MS cases^35^ and was demonstrated to stimulate a unique transcriptional signature in CD11b+/CD18+ microglia that activate a cascade of pro‐inflammatory events, including the recruitment and central nervous system (CNS) activation of myelin antigen‐specific Th1 cells and reactive oxygen species (ROS), TNF and IL1*β* release.^32^, ^34^, ^36^, ^37^ Chronification of these pathological events may contribute not only to tissue damage and neurodegeneration, but also to inhibition of potential repair mechanisms. Targeting the coagulation pathway might therefore have therapeutic potential.^38^ It still remains unclear how the whole fibrinogen molecule or some of the protein subunits may diffuse in CSF and brain tissue. The positive correlation that we found between albumin CSF/serum ratio and CSF levels of fibrinogen at the time of diagnosis suggests that blood/CSF/brain barrier alterations and/or BBB leakage may somehow influence intrathecal accumulation of molecules, potentially linked to periphery circulation, such as fibrinogen. On the contrary, the fact that similar correlations have not been detected between inflammatory mediators detected in the CSF and albumin CSF/serum ratio may suggest that CSF inflammatory markers are mainly associated with intrathecal inflammation, mediated by both CNS cells or intracerebral compartmentalized inflammation, as demonstrated by the strong correlation with CSF levels of sCD163. The significant correlation between fibrinogen and sCD163 supports the hypothesis that the CSF levels of the BBB disruption marker fibrinogen may also reflect to the degree of monocyte/macrophage activation and possibly to the presence of active pathological processes. In particular, it has been shown that the binding of the 377–395 amino acid portion of the *γ* chain fibrinogen molecule with the CD11b/CD18 receptor induces the activation of numerous intracellular pathways, including the activation of microglia with the production of a pro‐inflammatory milieu (cytokines and chemokines), able to recruit within the CNS monocytes and myelin antigen‐specific Th1 cells, and mediate axonal and neuronal damage^36^. The combined assessment of CSF levels of fibrinogen and sCD163 may, therefore, represent a potential tool to early investigate the activity stage of the lesions.

Studies on experimental autoimmune encephalomyelitis models suggested that upon BBB disruption, fibrinogen together with prothrombin may enter the CNS and local activation of thrombin may induce fibrin deposition since early stage of the disease^39^ (Davalos et al. Ann Neurol 2014). Therefore, complement and coagulation pathway possibly represents one of the earliest signs of inflammatory activity in lesions and its CSF examination may help to detect early pathological CNS injuries and neuroinflammation.

This study corroborates our previous suggestions that high CL load is associated with profound CSF inflammatory changes. In particular, CSF abundance of B cell‐linked inflammatory mediators, such as CXCL13, CXCL12, CXCL10, BAFF, IL6, IL10, GM‐CSF, and TNF, supports the hypothesis of a prominent role of B cells in the pathogenesis of CLs.^5^ These inflammatory mediators as well as one or more cyto‐ and/or myelinotoxic soluble CSF factors could then diffuse through the cortex and mediate a “surface‐inward” pattern of subpial cortical damage.^5^, ^7^, ^8^, ^9^, ^40^ A number of in vitro studies further support this hypothesis, showing that cultured neurons exposed to CSF of MS patients, but not of controls, undergo oxidative stress and axonal damage and that ceramides (C16:0 and C24:0) enriched in MS CSF patients may represent possible mediators of this injury.^41^, ^42^ In other works, mixed CNS glial cells exposed to supernatants of B cells isolated from MS patients but not from controls exhibited increased death of both oligodendrocytes^43^ and neurons.^44^ Moreover, monoclonal recombinant antibodies derived from expanded B‐cell clones isolated from CSF of MS patients induced demyelination and astrocyte activation on spinal cord explants.^45^

In addition, the CSF correlations found between B cell‐related molecules and macrophage markers may imply complex immunity interactions evocative of immunologic reactions occurring in the subcapsular sinus of lymph nodes or in the marginal zone of the spleen. These data suggest that CSF and meningeal infiltrating B cells might intrathecally interact with macrophages recognizing, internalizing, and retaining antigens through a variety of receptors including toll like receptors, C‐type lectin receptors but also scavenger receptors such as CD163.^46^ It remains to be clarified whether potential link between B‐cell immunity and complement‐coagulation cascade may indicate mechanisms of cortical damage  directly mediated by complement and/or opsonization .

This work may have some limitations: the proteomic analysis was performed only on a representative subset of MS patients. Considering that the applied mass spectrometry analysis was not quantitative, we performed the data validation with quantitative techniques (ELISA or Luminex experiments) involving a larger number of patients.

We suggest for the first time that intrathecal altered balance of complement/coagulation cascade, iron uptake, and innate response, in combination with dysregulated B‐cell immunity, may have a direct role in cortical damage since earliest MS stages and, possibly, in disease progression. Combined evaluation of CSF sCD14/sCD163 balance and of Hb/haptoglobin/fibrinogen levels, as well as of B‐cell activity biomarkers at the time of diagnosis may therefore represent a useful tool that, together with clinical and MRI assessment, may help to predict specific disease immunophenotypes.

### Study approval

The local Ethic Committee approved the study (Protocol number 35315, 31/07/2014). Written informed consent was obtained from each patient.

## Author Contributions

RM and MC contributed to the conception and design of the study; RM, SH, FF, DM, SR, and MC and MC contributed to the acquisition and analysis of data; RM, SH, DM, SM, HL, and MC contributed to interpretation of the results; RM, SH, DM, FF, SR, VM, FL, AV, RN, RR, SM, HL, and MC contributed to drafting and revision of the text.

## Conflict of Interest

The author(s) declare(s) that there is no conflict of interest.

## Supporting information


**Table S1.** Clinical parameters at the diagnosis of the control group.

**Table S2.** Clinical and MRI parameters at the diagnosis of the MS patients examined for CSF TRIDENT proteomic analysis.

**Table S3.** List of proteins and respective accession numbers detected by TRIDENT methodology followed by LC–MS/MS analysis.

**Table S4.** List of the 10 main pathways as key players differentiating the MShigh and MSlow patients

**Data S1.** Supplementary materials and methods.
