# Supplementary material for: Iron homeostasis, complement, and coagulation cascade as CSF signature of cortical lesions in early multiple sclerosis
Source: Ann Clin Transl Neurol. 2019 Nov 1;6(11):2150–63. doi: 10.1002/acn3.50893 (PMC6856609; doi:10.1002/acn3.50893)
Supplement: Supplementary file 1 — Table S1. Clinical parameters at the diagnosis of the control group. [file ACN3-6-2150-s001.doc]

Supplementary Table 1: Clinical parameters at the diagnosis of the control group.

|  | Total | Non-inflammatory neurological diseases (NIND) | Inflammatory neurological diseases (OIND) |
| --- | --- | --- | --- |
| Number | 26 | 12 | 14 |
| age | 46,2 + 10,1 | 45,5 + 10,04 | 46,7 + 11,5 |
| gender (f:m) | 16 / 10 | 9 / 7 | 8/ 6 |
| BOIgG positive/negative | All negative |  |  |

Age data are reported as mean ± standard deviation (range).

Non-inflammatory neurological diseases (NIND) include 3 acute cerebrovascular diseases, 3 degenerative disease, 2 peripheral neuropathies, 2 headache/dizziness without any CNS abnormality, 2 chronic ischemic cerebrovascular diseases; inflammatory neurological diseases (OIND) include 5 autoimmune encephalitis, 2 neuromyelitis optica spectrum disorders, 2 neurosarcoidosis, 2 idiophatic leucoencephalopathies, 2 primary CNS vascultis, 1 Behcet’s disease.
