# Supplementary material for: Iron homeostasis, complement, and coagulation cascade as CSF signature of cortical lesions in early multiple sclerosis
Source: Ann Clin Transl Neurol. 2019 Nov 1;6(11):2150–63. doi: 10.1002/acn3.50893 (PMC6856609; doi:10.1002/acn3.50893)
Supplement: Supplementary file 2 — Table S2. Clinical and MRI parameters at the diagnosis of the MS patients examined for CSF TRIDENT proteomic analysis. [file ACN3-6-2150-s002.docx]

Supplementary Table 2

| **MS patient** | **Age/Gender** | **EDSS at recruitment** | **OCB** | **IgG index** | **CLs volume** | **CLs number** | **T2WMLV** |
| --- | --- | --- | --- | --- | --- | --- | --- |
| **SM01** | 28/M | 2 | 1 | 0.5 | 540.0 | 3 | 4.6 |
| **SM12** | 34/F | 1.56 | 0 | 0.46 | 0 | 0 | 5.2 |
| **SM14** | 44/M | 3.5 | 1 | 0.97 | 105.0 | 1 | 6.3 |
| **SM02** | 29/F | 1 | 1 | 0.51 | 2156.0 | 22 | 4.3 |
| **SM13** | 41/F | 1 | 1 | 0.5 | 2668.9 | 29 | 2.0 |
| **SM57** | 63/M | 3 | 1 | 0.49 | 980.0 | 10 | 6.2 |

Abbreviations: EDSS: Expanded Disability Status Scale; OCB: oligoclonal bands; IgG index: immunoglobulin-G index; CLs: cortical lesions; T2WMLV: T2 white matter lesion volume.
