# Supplementary material for: Iron homeostasis, complement, and coagulation cascade as CSF signature of cortical lesions in early multiple sclerosis
Source: Ann Clin Transl Neurol. 2019 Nov 1;6(11):2150–63. doi: 10.1002/acn3.50893 (PMC6856609; doi:10.1002/acn3.50893)
Supplement: Supplementary file 3 — Table S3. List of proteins and respective accession numbers detected by TRIDENT methodology followed by LC–MS/MS analysis. [file ACN3-6-2150-s003.docx]

Supplementary Table 3. Proteomic analysis

| **N°** | **Accession** | **Description** |
| --- | --- | --- |
| **1** | **P31946** | **14-3-3 protein beta/alpha OS=Homo sapiens GN=YWHAB PE=1 SV=3 - [1433B_HUMAN]** |
| **2** | **P62258** | **14-3-3 protein epsilon OS=Homo sapiens GN=YWHAE PE=1 SV=1 - [1433E_HUMAN]** |
| **3** | **P31947** | **14-3-3 protein sigma OS=Homo sapiens GN=SFN PE=1 SV=1 - [1433S_HUMAN]** |
| **4** | **P63104** | **14-3-3 protein zeta/delta OS=Homo sapiens GN=YWHAZ PE=1 SV=1 - [1433Z_HUMAN]** |
| **5** | **P60709** | **Actin, cytoplasmic 1 OS=Homo sapiens GN=ACTB PE=1 SV=1 - [ACTB_HUMAN]** |
| **6** | **Q8IUX7** | **Adipocyte enhancer-binding protein 1 OS=Homo sapiens GN=AEBP1 PE=1 SV=1 - [AEBP1_HUMAN]** |
| **7** | **P43652** | **Afamin OS=Homo sapiens GN=AFM PE=1 SV=1 - [AFAM_HUMAN]** |
| **8** | **O00468** | **Agrin OS=Homo sapiens GN=AGRN PE=1 SV=5 - [AGRIN_HUMAN]** |
| **9** | **P02763** | **Alpha-1-acid glycoprotein 1 OS=Homo sapiens GN=ORM1 PE=1 SV=1 - [A1AG1_HUMAN]** |
| **10** | **P02763** | **Alpha-1-acid glycoprotein 1 OS=Homo sapiens GN=ORM1 PE=1 SV=1 - [A1AG1_HUMAN]** |
| **11** | **P19652** | **Alpha-1-acid glycoprotein 2 OS=Homo sapiens GN=ORM2 PE=1 SV=2 - [A1AG2_HUMAN]** |
| **12** | **P01011** | **Alpha-1-antichymotrypsin OS=Homo sapiens GN=SERPINA3 PE=1 SV=2 - [AACT_HUMAN]** |
| **13** | **P01009** | **Alpha-1-antitrypsin OS=Homo sapiens GN=SERPINA1 PE=1 SV=3 - [A1AT_HUMAN]** |
| **14** | **P04217** | **Alpha-1B-glycoprotein OS=Homo sapiens GN=A1BG PE=1 SV=4 - [A1BG_HUMAN]** |
| **15** | **P08697** | **Alpha-2-antiplasmin OS=Homo sapiens GN=SERPINF2 PE=1 SV=3 - [A2AP_HUMAN]** |
| **16** | **P02765** | **Alpha-2-HS-glycoprotein OS=Homo sapiens GN=AHSG PE=1 SV=1 - [FETUA_HUMAN]** |
| **17** | **P01023** | **Alpha-2-macroglobulin OS=Homo sapiens GN=A2M PE=1 SV=3 - [A2MG_HUMAN]** |
| **18** | **P12814** | **Alpha-actinin-1 OS=Homo sapiens GN=ACTN1 PE=1 SV=2 - [ACTN1_HUMAN]** |
| **19** | **P06733** | **Alpha-enolase OS=Homo sapiens GN=ENO1 PE=1 SV=2 - [ENOA_HUMAN]** |
| **20** | **P49641** | **Alpha-mannosidase 2x OS=Homo sapiens GN=MAN2A2 PE=2 SV=3 - [MA2A2_HUMAN]** |
| **21** | **P05067** | **Amyloid beta A4 protein OS=Homo sapiens GN=APP PE=1 SV=3 - [A4_HUMAN]** |
| **22** | **P51693** | **Amyloid-like protein 1 OS=Homo sapiens GN=APLP1 PE=1 SV=3 - [APLP1_HUMAN]** |
| **23** | **P01019** | **Angiotensinogen OS=Homo sapiens GN=AGT PE=1 SV=1 - [ANGT_HUMAN]** |
| **24** | **P04083** | **Annexin A1 OS=Homo sapiens GN=ANXA1 PE=1 SV=2 - [ANXA1_HUMAN]** |
| **25** | **P07355** | **Annexin A2 OS=Homo sapiens GN=ANXA2 PE=1 SV=2 - [ANXA2_HUMAN]** |
| **26** | **P01008** | **Antithrombin-III OS=Homo sapiens GN=SERPINC1 PE=1 SV=1 - [ANT3_HUMAN]** |
| **27** | **P02647** | **Apolipoprotein A-I OS=Homo sapiens GN=APOA1 PE=1 SV=1 - [APOA1_HUMAN]** |
| **28** | **P06727** | **Apolipoprotein A-IV OS=Homo sapiens GN=APOA4 PE=1 SV=3 - [APOA4_HUMAN]** |
| **29** | **P02649** | **Apolipoprotein E OS=Homo sapiens GN=APOE PE=1 SV=1 - [APOE_HUMAN]** |
| **30** | **P17174** | **Aspartate aminotransferase, cytoplasmic OS=Homo sapiens GN=GOT1 PE=1 SV=3 - [AATC_HUMAN]** |
| **31** | **P98160** | **Basement membrane-specific heparan sulfate proteoglycan core protein OS=Homo sapiens GN=HSPG2 PE=1 SV=4 - [PGBM_HUMAN]** |
| **32** | **O43505** | **Beta-1,4-glucuronyltransferase 1 OS=Homo sapiens GN=B4GAT1 PE=1 SV=1 - [B4GA1_HUMAN]** |
| **33** | **P02749** | **Beta-2-glycoprotein 1 OS=Homo sapiens GN=APOH PE=1 SV=3 - [APOH_HUMAN]** |
| **34** | **P61769** | **Beta-2-microglobulin OS=Homo sapiens GN=B2M PE=1 SV=1 - [B2MG_HUMAN]** |
| **35** | **Q96KN2** | **Beta-Ala-His dipeptidase OS=Homo sapiens GN=CNDP1 PE=1 SV=4 - [CNDP1_HUMAN]** |
| **36** | **P19022** | **Cadherin-2 OS=Homo sapiens GN=CDH2 PE=1 SV=4 - [CADH2_HUMAN]** |
| **37** | **Q9NZT1** | **Calmodulin-like protein 5 OS=Homo sapiens GN=CALML5 PE=1 SV=2 - [CALL5_HUMAN]** |
| **38** | **O94985** | **Calsyntenin-1 OS=Homo sapiens GN=CLSTN1 PE=1 SV=1 - [CSTN1_HUMAN]** |
| **39** | **P00915** | **Carbonic anhydrase 1 OS=Homo sapiens GN=CA1 PE=1 SV=2 - [CAH1_HUMAN]** |
| **40** | **P07451** | **Carbonic anhydrase 3 OS=Homo sapiens GN=CA3 PE=1 SV=3 - [CAH3_HUMAN]** |
| **41** | **P16870** | **Carboxypeptidase E OS=Homo sapiens GN=CPE PE=1 SV=1 - [CBPE_HUMAN]** |
| **42** | **Q9NQ79** | **Cartilage acidic protein 1 OS=Homo sapiens GN=CRTAC1 PE=1 SV=2 - [CRAC1_HUMAN]** |
| **43** | **P31944** | **Caspase-14 OS=Homo sapiens GN=CASP14 PE=1 SV=2 - [CASPE_HUMAN]** |
| **44** | **P07339** | **Cathepsin D OS=Homo sapiens GN=CTSD PE=1 SV=1 - [CATD_HUMAN]** |
| **45** | **P07711** | **Cathepsin L1 OS=Homo sapiens GN=CTSL PE=1 SV=2 - [CATL1_HUMAN]** |
| **46** | **P00450** | **Ceruloplasmin OS=Homo sapiens GN=CP PE=1 SV=1 - [CERU_HUMAN]** |
| **47** | **P36222** | **Chitinase-3-like protein 1 OS=Homo sapiens GN=CHI3L1 PE=1 SV=2 - [CH3L1_HUMAN]** |
| **48** | **P10909** | **Clusterin OS=Homo sapiens GN=CLU PE=1 SV=1 - [CLUS_HUMAN]** |
| **49** | **P12259** | **Coagulation factor V OS=Homo sapiens GN=F5 PE=1 SV=4 - [FA5_HUMAN]** |
| **50** | **P02452** | **Collagen alpha-1(I) chain OS=Homo sapiens GN=COL1A1 PE=1 SV=5 - [CO1A1_HUMAN]** |
| **51** | **P12109** | **Collagen alpha-1(VI) chain OS=Homo sapiens GN=COL6A1 PE=1 SV=3 - [CO6A1_HUMAN]** |
| **52** | **P02746** | **Complement C1q subcomponent subunit B OS=Homo sapiens GN=C1QB PE=1 SV=3 - [C1QB_HUMAN]** |
| **53** | **P00736** | **Complement C1r subcomponent OS=Homo sapiens GN=C1R PE=1 SV=2 - [C1R_HUMAN]** |
| **54** | **P09871** | **Complement C1s subcomponent OS=Homo sapiens GN=C1S PE=1 SV=1 - [C1S_HUMAN]** |
| **55** | **P06681** | **Complement C2 OS=Homo sapiens GN=C2 PE=1 SV=2 - [CO2_HUMAN]** |
| **56** | **P01024** | **Complement C3 OS=Homo sapiens GN=C3 PE=1 SV=2 - [CO3_HUMAN]** |
| **57** | **P0C0L4** | **Complement C4-A OS=Homo sapiens GN=C4A PE=1 SV=2 - [CO4A_HUMAN]** |
| **58** | **P0C0L5** | **Complement C4-B OS=Homo sapiens GN=C4B PE=1 SV=2 - [CO4B_HUMAN]** |
| **59** | **P01031** | **Complement C5 OS=Homo sapiens GN=C5 PE=1 SV=4 - [CO5_HUMAN]** |
| **60** | **P13671** | **Complement component C6 OS=Homo sapiens GN=C6 PE=1 SV=3 - [CO6_HUMAN]** |
| **61** | **P10643** | **Complement component C7 OS=Homo sapiens GN=C7 PE=1 SV=2 - [CO7_HUMAN]** |
| **62** | **P07360** | **Complement component C8 gamma chain OS=Homo sapiens GN=C8G PE=1 SV=3 - [CO8G_HUMAN]** |
| **63** | **P02748** | **Complement component C9 OS=Homo sapiens GN=C9 PE=1 SV=2 - [CO9_HUMAN]** |
| **64** | **P00751** | **Complement factor B OS=Homo sapiens GN=CFB PE=1 SV=2 - [CFAB_HUMAN]** |
| **65** | **P00746** | **Complement factor D OS=Homo sapiens GN=CFD PE=1 SV=5 - [CFAD_HUMAN]** |
| **66** | **P08603** | **Complement factor H OS=Homo sapiens GN=CFH PE=1 SV=4 - [CFAH_HUMAN]** |
| **67** | **P05156** | **Complement factor I OS=Homo sapiens GN=CFI PE=1 SV=2 - [CFAI_HUMAN]** |
| **68** | **Q12860** | **Contactin-1 OS=Homo sapiens GN=CNTN1 PE=1 SV=1 - [CNTN1_HUMAN]** |
| **69** | **Q02246** | **Contactin-2 OS=Homo sapiens GN=CNTN2 PE=1 SV=1 - [CNTN2_HUMAN]** |
| **70** | **Q8IWV2** | **Contactin-4 OS=Homo sapiens GN=CNTN4 PE=1 SV=1 - [CNTN4_HUMAN]** |
| **71** | **Q9C0A0** | **Contactin-associated protein-like 4 OS=Homo sapiens GN=CNTNAP4 PE=1 SV=3 - [CNTP4_HUMAN]** |
| **72** | **P08185** | **Corticosteroid-binding globulin OS=Homo sapiens GN=SERPINA6 PE=1 SV=1 - [CBG_HUMAN]** |
| **73** | **P01034** | **Cystatin-C OS=Homo sapiens GN=CST3 PE=1 SV=1 - [CYTC_HUMAN]** |
| **74** | **Q02413** | **Desmoglein-1 OS=Homo sapiens GN=DSG1 PE=1 SV=2 - [DSG1_HUMAN]** |
| **75** | **Q86SJ6** | **Desmoglein-4 OS=Homo sapiens GN=DSG4 PE=1 SV=1 - [DSG4_HUMAN]** |
| **76** | **P15924** | **Desmoplakin OS=Homo sapiens GN=DSP PE=1 SV=3 - [DESP_HUMAN]** |
| **77** | **Q9UBP4** | **Dickkopf-related protein 3 OS=Homo sapiens GN=DKK3 PE=1 SV=2 - [DKK3_HUMAN]** |
| **78** | **Q14118** | **Dystroglycan OS=Homo sapiens GN=DAG1 PE=1 SV=2 - [DAG1_HUMAN]** |
| **79** | **Q13822** | **Ectonucleotide pyrophosphatase/phosphodiesterase family member 2 OS=Homo sapiens GN=ENPP2 PE=1 SV=3 - [ENPP2_HUMAN]** |
| **80** | **Q12805** | **EGF-containing fibulin-like extracellular matrix protein 1 OS=Homo sapiens GN=EFEMP1 PE=1 SV=2 - [FBLN3_HUMAN]** |
| **81** | **P68104** | **Elongation factor 1-alpha 1 OS=Homo sapiens GN=EEF1A1 PE=1 SV=1 - [EF1A1_HUMAN]** |
| **82** | **O94919** | **Endonuclease domain-containing 1 protein OS=Homo sapiens GN=ENDOD1 PE=1 SV=2 - [ENDD1_HUMAN]** |
| **83** | **P61916** | **Epididymal secretory protein E1 OS=Homo sapiens GN=NPC2 PE=1 SV=1 - [NPC2_HUMAN]** |
| **84** | **Q16610** | **Extracellular matrix protein 1 OS=Homo sapiens GN=ECM1 PE=1 SV=2 - [ECM1_HUMAN]** |
| **85** | **P08294** | **Extracellular superoxide dismutase [Cu-Zn] OS=Homo sapiens GN=SOD3 PE=1 SV=2 - [SODE_HUMAN]** |
| **86** | **Q01469** | **Fatty acid-binding protein, epidermal OS=Homo sapiens GN=FABP5 PE=1 SV=3 - [FABP5_HUMAN]** |
| **87** | **P02671** | **Fibrinogen alpha chain OS=Homo sapiens GN=FGA PE=1 SV=2 - [FIBA_HUMAN]** |
| **88** | **P02675** | **Fibrinogen beta chain OS=Homo sapiens GN=FGB PE=1 SV=2 - [FIBB_HUMAN]** |
| **89** | **P02679** | **Fibrinogen gamma chain OS=Homo sapiens GN=FGG PE=1 SV=3 - [FIBG_HUMAN]** |
| **90** | **P02751** | **Fibronectin OS=Homo sapiens GN=FN1 PE=1 SV=4 - [FINC_HUMAN]** |
| **91** | **P23142** | **Fibulin-1 OS=Homo sapiens GN=FBLN1 PE=1 SV=4 - [FBLN1_HUMAN]** |
| **92** | **P98095** | **Fibulin-2 OS=Homo sapiens GN=FBLN2 PE=1 SV=2 - [FBLN2_HUMAN]** |
| **93** | **Q5D862** | **Filaggrin-2 OS=Homo sapiens GN=FLG2 PE=1 SV=1 - [FILA2_HUMAN]** |
| **94** | **Q08380** | **Galectin-3-binding protein OS=Homo sapiens GN=LGALS3BP PE=1 SV=1 - [LG3BP_HUMAN]** |
| **95** | **P17900** | **Ganglioside GM2 activator OS=Homo sapiens GN=GM2A PE=1 SV=4 - [SAP3_HUMAN]** |
| **96** | **P06396** | **Gelsolin OS=Homo sapiens GN=GSN PE=1 SV=1 - [GELS_HUMAN]** |
| **97** | **P15104** | **Glutamine synthetase OS=Homo sapiens GN=GLUL PE=1 SV=4 - [GLNA_HUMAN]** |
| **98** | **Q16769** | **Glutaminyl-peptide cyclotransferase OS=Homo sapiens GN=QPCT PE=1 SV=1 - [QPCT_HUMAN]** |
| **99** | **P22352** | **Glutathione peroxidase 3 OS=Homo sapiens GN=GPX3 PE=1 SV=2 - [GPX3_HUMAN]** |
| **100** | **P04406** | **Glyceraldehyde-3-phosphate dehydrogenase OS=Homo sapiens GN=GAPDH PE=1 SV=3 - [G3P_HUMAN]** |
| **101** | **P00738** | **Haptoglobin OS=Homo sapiens GN=HP PE=1 SV=1 - [HPT_HUMAN]** |
| **102** | **P11142** | **Heat shock cognate 71 kDa protein OS=Homo sapiens GN=HSPA8 PE=1 SV=1 - [HSP7C_HUMAN]** |
| **103** | **P69905** | **Hemoglobin subunit alpha OS=Homo sapiens GN=HBA1 PE=1 SV=2 - [HBA_HUMAN]** |
| **104** | **P68871** | **Hemoglobin subunit beta OS=Homo sapiens GN=HBB PE=1 SV=2 - [HBB_HUMAN]** |
| **105** | **P02790** | **Hemopexin OS=Homo sapiens GN=HPX PE=1 SV=2 - [HEMO_HUMAN]** |
| **106** | **P05546** | **Heparin cofactor 2 OS=Homo sapiens GN=SERPIND1 PE=1 SV=3 - [HEP2_HUMAN]** |
| **107** | **P04196** | **Histidine-rich glycoprotein OS=Homo sapiens GN=HRG PE=1 SV=1 - [HRG_HUMAN]** |
| **108** | **P22304** | **Iduronate 2-sulfatase OS=Homo sapiens GN=IDS PE=1 SV=1 - [IDS_HUMAN]** |
| **109** | **P01876** | **Ig alpha-1 chain C region OS=Homo sapiens GN=IGHA1 PE=1 SV=2 - [IGHA1_HUMAN]** |
| **110** | **P01877** | **Ig alpha-2 chain C region OS=Homo sapiens GN=IGHA2 PE=1 SV=3 - [IGHA2_HUMAN]** |
| **111** | **P01857** | **Ig gamma-1 chain C region OS=Homo sapiens GN=IGHG1 PE=1 SV=1 - [IGHG1_HUMAN]** |
| **112** | **P01859** | **Ig gamma-2 chain C region OS=Homo sapiens GN=IGHG2 PE=1 SV=2 - [IGHG2_HUMAN]** |
| **113** | **P01860** | **Ig gamma-3 chain C region OS=Homo sapiens GN=IGHG3 PE=1 SV=2 - [IGHG3_HUMAN]** |
| **114** | **P01861** | **Ig gamma-4 chain C region OS=Homo sapiens GN=IGHG4 PE=1 SV=1 - [IGHG4_HUMAN]** |
| **115** | **P01766** | **Ig heavy chain V-III region BRO OS=Homo sapiens PE=1 SV=1 - [HV305_HUMAN]** |
| **116** | **P01781** | **Ig heavy chain V-III region GAL OS=Homo sapiens PE=1 SV=1 - [HV320_HUMAN]** |
| **117** | **P01779** | **Ig heavy chain V-III region TUR OS=Homo sapiens PE=1 SV=1 - [HV318_HUMAN]** |
| **118** | **P01834** | **Ig kappa chain C region OS=Homo sapiens GN=IGKC PE=1 SV=1 - [IGKC_HUMAN]** |
| **119** | **P01593** | **Ig kappa chain V-I region AG OS=Homo sapiens PE=1 SV=1 - [KV101_HUMAN]** |
| **120** | **P01594** | **Ig kappa chain V-I region AU OS=Homo sapiens PE=1 SV=1 - [KV102_HUMAN]** |
| **121** | **P01613** | **Ig kappa chain V-I region Ni OS=Homo sapiens PE=1 SV=1 - [KV121_HUMAN]** |
| **122** | **P01608** | **Ig kappa chain V-I region Roy OS=Homo sapiens PE=1 SV=1 - [KV116_HUMAN]** |
| **123** | **P01617** | **Ig kappa chain V-II region TEW OS=Homo sapiens PE=1 SV=1 - [KV204_HUMAN]** |
| **124** | **P18135** | **Ig kappa chain V-III region HAH OS=Homo sapiens PE=2 SV=1 - [KV312_HUMAN]** |
| **125** | **P01620** | **Ig kappa chain V-III region SIE OS=Homo sapiens PE=1 SV=1 - [KV302_HUMAN]** |
| **126** | **P01625** | **Ig kappa chain V-IV region Len OS=Homo sapiens PE=1 SV=2 - [KV402_HUMAN]** |
| **127** | **P80748** | **Ig lambda chain V-III region LOI OS=Homo sapiens PE=1 SV=1 - [LV302_HUMAN]** |
| **128** | **P0CG04** | **Ig lambda-1 chain C regions OS=Homo sapiens GN=IGLC1 PE=1 SV=1 - [LAC1_HUMAN]** |
| **129** | **P0CG05** | **Ig lambda-2 chain C regions OS=Homo sapiens GN=IGLC2 PE=1 SV=1 - [LAC2_HUMAN]** |
| **130** | **A0M8Q6** | **Ig lambda-7 chain C region OS=Homo sapiens GN=IGLC7 PE=4 SV=2 - [LAC7_HUMAN]** |
| **131** | **P01871** | **Ig mu chain C region OS=Homo sapiens GN=IGHM PE=1 SV=3 - [IGHM_HUMAN]** |
| **132** | **P04220** | **Ig mu heavy chain disease protein OS=Homo sapiens PE=1 SV=1 - [MUCB_HUMAN]** |
| **133** | **Q9Y6R7** | **IgGFc-binding protein OS=Homo sapiens GN=FCGBP PE=1 SV=3 - [FCGBP_HUMAN]** |
| **134** | **P18065** | **Insulin-like growth factor-binding protein 2 OS=Homo sapiens GN=IGFBP2 PE=1 SV=2 - [IBP2_HUMAN]** |
| **135** | **Q16270** | **Insulin-like growth factor-binding protein 7 OS=Homo sapiens GN=IGFBP7 PE=1 SV=1 - [IBP7_HUMAN]** |
| **136** | **P19827** | **Inter-alpha-trypsin inhibitor heavy chain H1 OS=Homo sapiens GN=ITIH1 PE=1 SV=3 - [ITIH1_HUMAN]** |
| **137** | **P19823** | **Inter-alpha-trypsin inhibitor heavy chain H2 OS=Homo sapiens GN=ITIH2 PE=1 SV=2 - [ITIH2_HUMAN]** |
| **138** | **Q14624** | **Inter-alpha-trypsin inhibitor heavy chain H4 OS=Homo sapiens GN=ITIH4 PE=1 SV=4 - [ITIH4_HUMAN]** |
| **139** | **P14923** | **Junction plakoglobin OS=Homo sapiens GN=JUP PE=1 SV=3 - [PLAK_HUMAN]** |
| **140** | **Q92876** | **Kallikrein-6 OS=Homo sapiens GN=KLK6 PE=1 SV=1 - [KLK6_HUMAN]** |
| **141** | **Q5T749** | **Keratinocyte proline-rich protein OS=Homo sapiens GN=KPRP PE=1 SV=1 - [KPRP_HUMAN]** |
| **142** | **P01042** | **Kininogen-1 OS=Homo sapiens GN=KNG1 PE=1 SV=2 - [KNG1_HUMAN]** |
| **143** | **P11047** | **Laminin subunit gamma-1 OS=Homo sapiens GN=LAMC1 PE=1 SV=3 - [LAMC1_HUMAN]** |
| **144** | **Q8N2S1** | **Latent-transforming growth factor beta-binding protein 4 OS=Homo sapiens GN=LTBP4 PE=1 SV=2 - [LTBP4_HUMAN]** |
| **145** | **Q9NT99** | **Leucine-rich repeat-containing protein 4B OS=Homo sapiens GN=LRRC4B PE=2 SV=3 - [LRC4B_HUMAN]** |
| **146** | **P07195** | **L-lactate dehydrogenase B chain OS=Homo sapiens GN=LDHB PE=1 SV=2 - [LDHB_HUMAN]** |
| **147** | **P51884** | **Lumican OS=Homo sapiens GN=LUM PE=1 SV=2 - [LUM_HUMAN]** |
| **148** | **P61626** | **Lysozyme C OS=Homo sapiens GN=LYZ PE=1 SV=1 - [LYSC_HUMAN]** |
| **149** | **P07333** | **Macrophage colony-stimulating factor 1 receptor OS=Homo sapiens GN=CSF1R PE=1 SV=2 - [CSF1R_HUMAN]** |
| **150** | **P20774** | **Mimecan OS=Homo sapiens GN=OGN PE=1 SV=1 - [MIME_HUMAN]** |
| **151** | **P08571** | **Monocyte differentiation antigen CD14 OS=Homo sapiens GN=CD14 PE=1 SV=2 - [CD14_HUMAN]** |
| **152** | **Q7Z7M0** | **Multiple epidermal growth factor-like domains protein 8 OS=Homo sapiens GN=MEGF8 PE=1 SV=2 - [MEGF8_HUMAN]** |
| **153** | **P12883** | **Myosin-7 OS=Homo sapiens GN=MYH7 PE=1 SV=5 - [MYH7_HUMAN]** |
| **154** | **Q96PD5** | **N-acetylmuramoyl-L-alanine amidase OS=Homo sapiens GN=PGLYRP2 PE=1 SV=1 - [PGRP2_HUMAN]** |
| **155** | **Q92859** | **Neogenin OS=Homo sapiens GN=NEO1 PE=1 SV=2 - [NEO1_HUMAN]** |
| **156** | **P13591** | **Neural cell adhesion molecule 1 OS=Homo sapiens GN=NCAM1 PE=1 SV=3 - [NCAM1_HUMAN]** |
| **157** | **O15394** | **Neural cell adhesion molecule 2 OS=Homo sapiens GN=NCAM2 PE=1 SV=2 - [NCAM2_HUMAN]** |
| **158** | **O00533** | **Neural cell adhesion molecule L1-like protein OS=Homo sapiens GN=CHL1 PE=1 SV=4 - [NCHL1_HUMAN]** |
| **159** | **Q9ULB1** | **Neurexin-1 OS=Homo sapiens GN=NRXN1 PE=2 SV=1 - [NRX1A_HUMAN]** |
| **160** | **Q9Y4C0** | **Neurexin-3 OS=Homo sapiens GN=NRXN3 PE=1 SV=4 - [NRX3A_HUMAN]** |
| **161** | **O14594** | **Neurocan core protein OS=Homo sapiens GN=NCAN PE=1 SV=3 - [NCAN_HUMAN]** |
| **162** | **O94856** | **Neurofascin OS=Homo sapiens GN=NFASC PE=1 SV=4 - [NFASC_HUMAN]** |
| **163** | **Q92823** | **Neuronal cell adhesion molecule OS=Homo sapiens GN=NRCAM PE=1 SV=3 - [NRCAM_HUMAN]** |
| **164** | **O95502** | **Neuronal pentraxin receptor OS=Homo sapiens GN=NPTXR PE=3 SV=2 - [NPTXR_HUMAN]** |
| **165** | **Q15818** | **Neuronal pentraxin-1 OS=Homo sapiens GN=NPTX1 PE=2 SV=2 - [NPTX1_HUMAN]** |
| **166** | **Q99574** | **Neuroserpin OS=Homo sapiens GN=SERPINI1 PE=1 SV=1 - [NEUS_HUMAN]** |
| **167** | **P10451** | **Osteopontin OS=Homo sapiens GN=SPP1 PE=1 SV=1 - [OSTP_HUMAN]** |
| **168** | **P26022** | **Pentraxin-related protein PTX3 OS=Homo sapiens GN=PTX3 PE=1 SV=3 - [PTX3_HUMAN]** |
| **169** | **Q6UXB8** | **Peptidase inhibitor 16 OS=Homo sapiens GN=PI16 PE=1 SV=1 - [PI16_HUMAN]** |
| **170** | **P19021** | **Peptidyl-glycine alpha-amidating monooxygenase OS=Homo sapiens GN=PAM PE=1 SV=2 - [AMD_HUMAN]** |
| **171** | **O60437** | **Periplakin OS=Homo sapiens GN=PPL PE=1 SV=4 - [PEPL_HUMAN]** |
| **172** | **P30086** | **Phosphatidylethanolamine-binding protein 1 OS=Homo sapiens GN=PEBP1 PE=1 SV=3 - [PEBP1_HUMAN]** |
| **173** | **Q96S96** | **Phosphatidylethanolamine-binding protein 4 OS=Homo sapiens GN=PEBP4 PE=1 SV=3 - [PEBP4_HUMAN]** |
| **174** | **P55058** | **Phospholipid transfer protein OS=Homo sapiens GN=PLTP PE=1 SV=1 - [PLTP_HUMAN]** |
| **175** | **P36955** | **Pigment epithelium-derived factor OS=Homo sapiens GN=SERPINF1 PE=1 SV=4 - [PEDF_HUMAN]** |
| **176** | **P05155** | **Plasma protease C1 inhibitor OS=Homo sapiens GN=SERPING1 PE=1 SV=2 - [IC1_HUMAN]** |
| **177** | **P05154** | **Plasma serine protease inhibitor OS=Homo sapiens GN=SERPINA5 PE=1 SV=3 - [IPSP_HUMAN]** |
| **178** | **P00747** | **Plasminogen OS=Homo sapiens GN=PLG PE=1 SV=2 - [PLMN_HUMAN]** |
| **179** | **P0CG48** | **Polyubiquitin-C OS=Homo sapiens GN=UBC PE=1 SV=3 - [UBC_HUMAN]** |
| **180** | **Q15113** | **Procollagen C-endopeptidase enhancer 1 OS=Homo sapiens GN=PCOLCE PE=1 SV=2 - [PCOC1_HUMAN]** |
| **181** | **Q9UHG2** | **ProSAAS OS=Homo sapiens GN=PCSK1N PE=1 SV=1 - [PCSK1_HUMAN]** |
| **182** | **P41222** | **Prostaglandin-H2 D-isomerase OS=Homo sapiens GN=PTGDS PE=1 SV=1 - [PTGDS_HUMAN]** |
| **183** | **Q9NY61** | **Protein AATF OS=Homo sapiens GN=AATF PE=1 SV=1 - [AATF_HUMAN]** |
| **184** | **P02760** | **Protein AMBP OS=Homo sapiens GN=AMBP PE=1 SV=1 - [AMBP_HUMAN]** |
| **185** | **P02760** | **Protein AMBP OS=Homo sapiens GN=AMBP PE=1 SV=1 - [AMBP_HUMAN]** |
| **186** | **Q92520** | **Protein FAM3C OS=Homo sapiens GN=FAM3C PE=1 SV=1 - [FAM3C_HUMAN]** |
| **187** | **Q99435** | **Protein kinase C-binding protein NELL2 OS=Homo sapiens GN=NELL2 PE=1 SV=1 - [NELL2_HUMAN]** |
| **188** | **P31151** | **Protein S100-A7 OS=Homo sapiens GN=S100A7 PE=1 SV=4 - [S10A7_HUMAN]** |
| **189** | **P06702** | **Protein S100-A9 OS=Homo sapiens GN=S100A9 PE=1 SV=1 - [S10A9_HUMAN]** |
| **190** | **P00734** | **Prothrombin OS=Homo sapiens GN=F2 PE=1 SV=2 - [THRB_HUMAN]** |
| **191** | **Q9NYQ8** | **Protocadherin Fat 2 OS=Homo sapiens GN=FAT2 PE=1 SV=2 - [FAT2_HUMAN]** |
| **192** | **A6NCN2** | **Putative keratin-87 protein OS=Homo sapiens GN=KRT87P PE=5 SV=4 - [KR87P_HUMAN]** |
| **193** | **P78509** | **Reelin OS=Homo sapiens GN=RELN PE=1 SV=3 - [RELN_HUMAN]** |
| **194** | **Q99969** | **Retinoic acid receptor responder protein 2 OS=Homo sapiens GN=RARRES2 PE=1 SV=1 - [RARR2_HUMAN]** |
| **195** | **P02753** | **Retinol-binding protein 4 OS=Homo sapiens GN=RBP4 PE=1 SV=3 - [RET4_HUMAN]** |
| **196** | **P34096** | **Ribonuclease 4 OS=Homo sapiens GN=RNASE4 PE=1 SV=3 - [RNAS4_HUMAN]** |
| **197** | **P05060** | **Secretogranin-1 OS=Homo sapiens GN=CHGB PE=1 SV=2 - [SCG1_HUMAN]** |
| **198** | **Q8WXD2** | **Secretogranin-3 OS=Homo sapiens GN=SCG3 PE=1 SV=3 - [SCG3_HUMAN]** |
| **199** | **Q53EL9** | **Seizure protein 6 homolog OS=Homo sapiens GN=SEZ6 PE=1 SV=2 - [SEZ6_HUMAN]** |
| **200** | **O75326** | **Semaphorin-7A OS=Homo sapiens GN=SEMA7A PE=1 SV=1 - [SEM7A_HUMAN]** |
| **201** | **P02787** | **Serotransferrin OS=Homo sapiens GN=TF PE=1 SV=3 - [TRFE_HUMAN]** |
| **202** | **P29508** | **Serpin B3 OS=Homo sapiens GN=SERPINB3 PE=1 SV=2 - [SPB3_HUMAN]** |
| **203** | **P48594** | **Serpin B4 OS=Homo sapiens GN=SERPINB4 PE=1 SV=2 - [SPB4_HUMAN]** |
| **204** | **P36952** | **Serpin B5 OS=Homo sapiens GN=SERPINB5 PE=1 SV=2 - [SPB5_HUMAN]** |
| **205** | **P02768** | **Serum albumin OS=Homo sapiens GN=ALB PE=1 SV=2 - [ALBU_HUMAN]** |
| **206** | **Q96PX8** | **SLIT and NTRK-like protein 1 OS=Homo sapiens GN=SLITRK1 PE=1 SV=2 - [SLIK1_HUMAN]** |
| **207** | **Q14515** | **SPARC-like protein 1 OS=Homo sapiens GN=SPARCL1 PE=1 SV=2 - [SPRL1_HUMAN]** |
| **208** | **O00391** | **Sulfhydryl oxidase 1 OS=Homo sapiens GN=QSOX1 PE=1 SV=3 - [QSOX1_HUMAN]** |
| **209** | **P00441** | **Superoxide dismutase [Cu-Zn] OS=Homo sapiens GN=SOD1 PE=1 SV=2 - [SODC_HUMAN]** |
| **210** | **P05452** | **Tetranectin OS=Homo sapiens GN=CLEC3B PE=1 SV=3 - [TETN_HUMAN]** |
| **211** | **P04216** | **Thy-1 membrane glycoprotein OS=Homo sapiens GN=THY1 PE=1 SV=2 - [THY1_HUMAN]** |
| **212** | **P55072** | **Transitional endoplasmic reticulum ATPase OS=Homo sapiens GN=VCP PE=1 SV=4 - [TERA_HUMAN]** |
| **213** | **Q24JP5** | **Transmembrane protein 132A OS=Homo sapiens GN=TMEM132A PE=1 SV=1 - [T132A_HUMAN]** |
| **214** | **P60174** | **Triosephosphate isomerase OS=Homo sapiens GN=TPI1 PE=1 SV=3 - [TPIS_HUMAN]** |
| **215** | **P07477** | **Trypsin-1 OS=Homo sapiens GN=PRSS1 PE=1 SV=1 - [TRY1_HUMAN]** |
| **216** | **Q9BQE3** | **Tubulin alpha-1C chain OS=Homo sapiens GN=TUBA1C PE=1 SV=1 - [TBA1C_HUMAN]** |
| **217** | **P68366** | **Tubulin alpha-4A chain OS=Homo sapiens GN=TUBA4A PE=1 SV=1 - [TBA4A_HUMAN]** |
| **218** | **P68371** | **Tubulin beta-4B chain OS=Homo sapiens GN=TUBB4B PE=1 SV=1 - [TBB4B_HUMAN]** |
| **219** | **Q6UX73** | **UPF0764 protein C16orf89 OS=Homo sapiens GN=C16orf89 PE=1 SV=2 - [CP089_HUMAN]** |
| **220** | **P19320** | **Vascular cell adhesion protein 1 OS=Homo sapiens GN=VCAM1 PE=1 SV=1 - [VCAM1_HUMAN]** |
| **221** | **Q6EMK4** | **Vasorin OS=Homo sapiens GN=VASN PE=1 SV=1 - [VASN_HUMAN]** |
| **222** | **Q12907** | **Vesicular integral-membrane protein VIP36 OS=Homo sapiens GN=LMAN2 PE=1 SV=1 - [LMAN2_HUMAN]** |
| **223** | **P02774** | **Vitamin D-binding protein OS=Homo sapiens GN=GC PE=1 SV=1 - [VTDB_HUMAN]** |
| **224** | **P04004** | **Vitronectin OS=Homo sapiens GN=VTN PE=1 SV=1 - [VTNC_HUMAN]** |
| **225** | **P54289** | **Voltage-dependent calcium channel subunit alpha-2/delta-1 OS=Homo sapiens GN=CACNA2D1 PE=1 SV=3 - [CA2D1_HUMAN]** |
| **226** | **Q15904** | **V-type proton ATPase subunit S1 OS=Homo sapiens GN=ATP6AP1 PE=1 SV=2 - [VAS1_HUMAN]** |
| **227** | **P25311** | **Zinc-alpha-2-glycoprotein OS=Homo sapiens GN=AZGP1 PE=1 SV=2 - [ZA2G_HUMAN]** |

Supplementary Table 3 List of proteins and respective accession numbers detected by TRIDENT metodology followed by LC-MS/MS analysis.
