# Supplementary material for: Iron homeostasis, complement, and coagulation cascade as CSF signature of cortical lesions in early multiple sclerosis
Source: Ann Clin Transl Neurol. 2019 Nov 1;6(11):2150–63. doi: 10.1002/acn3.50893 (PMC6856609; doi:10.1002/acn3.50893)
Supplement: Supplementary file 4 — Table S4. List of the 10 main pathways as key players differentiating the MShigh and MSlow patients [file ACN3-6-2150-s004.docx]

**Supplementary Table 4**

List of the 10 main pathways as key players differentiating the MShigh and MSlow patients

|  |  |
| --- | --- |
| **Protein list** | **Pathway** |
| **1** | **Complement activation, regulation of complement activation** |
| P10909 | clusterin(CLU) |
| P09871 | complement C1s(C1S) |
| P01024 | complement C3(C3) |
| P0C0L4 | complement C4A (Rodgers blood group)(C4A) |
| P0C0L5 | complement C4B (Chido blood group)(C4B) |
| P01031 | complement C5(C5) |
| P10643 | complement C7(C7) |
| P00751 | complement factor B(CFB) |
| P01857 | immunoglobulin heavy constant gamma 1 (G1m marker)(IGHG1) |
| P01859 | immunoglobulin heavy constant gamma 2 (G2m marker)(IGHG2) |
| P01860 | immunoglobulin heavy constant gamma 3 (G3m marker)(IGHG3) |
| P01861 | immunoglobulin heavy constant gamma 4 (G4m marker)(IGHG4) |
| P01834 | immunoglobulin kappa constant(IGKC) |
| P01620 | immunoglobulin kappa variable 3-20(IGKV3-20) |
| P0CG04 | immunoglobulin lambda constant 1(IGLC1) |
|  |  |
| **2** | [**Receptor-mediated endocytosis**](http://www.ebi.ac.uk/QuickGO/GTerm?id=GO:0006898) |
| P08571 | CD14 molecule(CD14) |
| P02768 | albumin(ALB) |
| P02760 | alpha-1-microglobulin/bikunin precursor(AMBP) |
| P02647 | apolipoprotein A1(APOA1) |
| P02649 | apolipoprotein E(APOE) |
| P05156 | complement factor I(CFI) |
| Q13822 | ectonucleotide pyrophosphatase/phosphodiesterase 2(ENPP2) |
| Q08380 | galectin 3 binding protein(LGALS3BP) |
| P00738 | haptoglobin(HP) |
| P69905 | hemoglobin subunit alpha 1(HBA1) |
| P68871 | hemoglobin subunit beta(HBB) |
| P02790 | hemopexin(HPX) |
| P01876 | immunoglobulin heavy constant alpha 1(IGHA1) |
| P01877 | immunoglobulin heavy constant alpha 2 (A2m marker)(IGHA2) |
| P01766 | immunoglobulin heavy variable 3-13(IGHV3-13) |
| P01779 | immunoglobulin heavy variable 3-23(IGHV3-23) |
| P01781 | immunoglobulin heavy variable 3-7(IGHV3-7) |
| P01834 | immunoglobulin kappa constant(IGKC) |
| P01593, P01608, P01613, P01594 | immunoglobulin kappa variable 1D-33(IGKV1D-33) |
| P01617 | immunoglobulin kappa variable 2D-28(IGKV2D-28) |
| P01620, P18135 | immunoglobulin kappa variable 3-20(IGKV3-20) |
| P01625 | immunoglobulin kappa variable 4-1(IGKV4-1) |
| P0CG04 | immunoglobulin lambda constant 1(IGLC1) |
| P0CG05 | immunoglobulin lambda constant 2(IGLC2) |
| A0M8Q6 | immunoglobulin lambda constant 7(IGLC7) |
| P80748 | immunoglobulin lambda variable 3-21(IGLV3-21) |
| P04004 | vitronectin(VTN) |
|  |  |
| **3** | **Innate immune response** |
| P10909 | clusterin(CLU) |
| P09871 | complement C1s(C1S) |
| P0C0L4 | complement C4A (Rodgers blood group)(C4A) |
| P0C0L5 | complement C4B (Chido blood group)(C4B) |
| P02675 | fibrinogen beta chain(FGB) |
| P01857 | immunoglobulin heavy constant gamma 1 (G1m marker)(IGHG1) |
| P01859 | immunoglobulin heavy constant gamma 2 (G2m marker)(IGHG2) |
| P01860 | immunoglobulin heavy constant gamma 3 (G3m marker)(IGHG3) |
| P01861 | immunoglobulin heavy constant gamma 4 (G4m marker)(IGHG4) |
| P01834 | immunoglobulin kappa constant(IGKC) |
| P0CG04 | immunoglobulin lambda constant 1(IGLC1) |
| P05155 | serpin family G member 1(SERPING1) |
|  |  |
| **4** | **Positive regulation of B cell activation, Bcell receptor signaling pathways** |
| P01857 | immunoglobulin heavy constant gamma 1 (G1m marker)(IGHG1) |
| P01859 | immunoglobulin heavy constant gamma 2 (G2m marker)(IGHG2) |
| P01860 | immunoglobulin heavy constant gamma 3 (G3m marker)(IGHG3) |
| P01861 | immunoglobulin heavy constant gamma 4 (G4m marker)(IGHG4) |
| P01834 | immunoglobulin kappa constant(IGKC) |
| P0CG04 | immunoglobulin lambda constant 1(IGLC1) |
|  |  |
| **5** | **Phagocytosis, recognition** |
| P01857 | immunoglobulin heavy constant gamma 1 (G1m marker)(IGHG1) |
| P01859 | immunoglobulin heavy constant gamma 2 (G2m marker)(IGHG2) |
| P01860 | immunoglobulin heavy constant gamma 3 (G3m marker)(IGHG3) |
| P01861 | immunoglobulin heavy constant gamma 4 (G4m marker)(IGHG4) |
| P01834 | immunoglobulin kappa constant(IGKC) |
| P0CG04 | immunoglobulin lambda constant 1(IGLC1) |
|  |  |
| **6** | **Fibrinolysis** |
| P02675 | fibrinogen beta chain(FGB) |
| P04196 | histidine rich glycoprotein(HRG) |
| P04264 | keratin 1(KRT1) |
| P05155 | serpin family G member 1(SERPING1) |
|  |  |
| **7** | **Fc-gamma receptor signaling pathway involved in phagocytosis** |
| P01857 | immunoglobulin heavy constant gamma 1 (G1m marker)(IGHG1) |
| P01859 | immunoglobulin heavy constant gamma 2 (G2m marker)(IGHG2) |
| P01860 | immunoglobulin heavy constant gamma 3 (G3m marker)(IGHG3) |
| P01861 | immunoglobulin heavy constant gamma 4 (G4m marker)(IGHG4) |
| P01834 | immunoglobulin kappa constant(IGKC) |
| P01620 | immunoglobulin kappa variable 3-20(IGKV3-20) |
| P0CG04 | immunoglobulin lambda constant 1(IGLC1) |
|  |  |
| **8** | [**Acute-phase response**](http://www.ebi.ac.uk/QuickGO/GTerm?id=GO:0006953) |
| P02765 | alpha 2-HS glycoprotein(AHSG) |
| P02751 | fibronectin 1(FN1) |
| P00738 | haptoglobin(HP) |
| Q14624 | inter-alpha-trypsin inhibitor heavy chain family member 4(ITIH4) |
| P02763 | orosomucoid 1(ORM1) |
| P19652 | orosomucoid 2(ORM2) |
| P01009 | serpin family A member 1(SERPINA1) |
| P01011 | serpin family A member 3(SERPINA3) |
|  |  |
| **9** | **Cellular iron ion homeostasis** |
| P00450 | ceruloplasmin(CP) |
| P02790 | hemopexin(HPX) |
| P02787 | transferrin(TF) |
| P00738 | haptoglobin(HP) |
| P69905 | hemoglobin subunit alpha 1(HBA1) |
| P68871 | hemoglobin subunit beta(HBB) |
|  |  |
| **10** | **Negative regulation of blood coagulation** |
| P01024 | [complement C3(C3)](https://david.ncifcrf.gov/geneReportFull.jsp?rowids=718) |
| P0C0L4 | [complement C4A (Rodgers blood group)(C4A)](https://david.ncifcrf.gov/geneReportFull.jsp?rowids=720) |
| P0C0L5 | [complement C4B (Chido blood group)(C4B)](https://david.ncifcrf.gov/geneReportFull.jsp?rowids=721) |
| P01031 | [complement C5(C5)](https://david.ncifcrf.gov/geneReportFull.jsp?rowids=727) |
| P02763 | [orosomucoid 1(ORM1)](https://david.ncifcrf.gov/geneReportFull.jsp?rowids=5004) |
| P01011 | [serpin family A member 3(SERPINA3)](https://david.ncifcrf.gov/geneReportFull.jsp?rowids=12) |
| P08571 | CD14 molecule(CD14) |
| P02675 | Fibrinogen beta chain(FBG) |
