# Supplementary material for: Iron homeostasis, complement, and coagulation cascade as CSF signature of cortical lesions in early multiple sclerosis
Source: Ann Clin Transl Neurol. 2019 Nov 1;6(11):2150–63. doi: 10.1002/acn3.50893 (PMC6856609; doi:10.1002/acn3.50893)
Supplement: Supplementary file 5 — Data S1. Supplementary materials and methods. [file ACN3-6-2150-s005.docx]

**Supplementary materials and methods**

**MRI Acquisition Protocol and analysis**

In each patient, MRI was performed at least 2 months after the last relapse. MRI sequences were acquired using a Philips Achieva 3T MR Scanner. The following image sets were acquired: in each patient, MRI was performed at least 2 months after the last relapse. MRI sequences were acquired using a Philips Achieva 3T MR Scanner. The following image sets were acquired: a) 3D T1 weighted Turbo Field Echo (TFE) (Repetition Time (TR) / Echo Time (TE) = 8.4/3.7ms, voxel size of 1x1x1 mm), Total acquisition time of 5:51 min; b) 3D Double Inversion Recovery (DIR) (TR/TE = 5500/292ms, Inversion Times (TI) TI1/TI2 = 525ms/2530ms voxel size of 1x1x1mm), Turbo Spin Echo (TSE) total acquisition time of 10:49 min; c) 3D Fluid Attenuated Inversion Recovery (FLAIR) (TR/TE = 5500/292ms, TI = 1650ms voxel size of 1x1x1mm), same TSE readout as the DIR sequence, total acquisition time of 5:44 min. Optimized parameters of each sequence were set as previously reported.^1^

**MRI analysis**

**a) WM and CL lesion detection and lesion load assessment**

White matter lesions were assessed by consensus of experienced observers, as previously described.^2^ Attention was paid to manually check the lesion map to identify and segment WM lesions, thus obtaining a T2-hyperintense WM lesion volume (T2WMLV). A similar consensus procedure was followed for CLs detection on DIR images, following recommendations for CLs scoring in patients with MS.^3^ Moreover, CLs were sub-divided in leukocortical and intracortical based on visual assessment and the cortical lesion volume was obtained as previously described.^4^

**b) Cortical thickness evaluation and T1 parcellation**

The estimation of the cortical thickness was performed using the 3D T1 sequence and the automated, volume-based Advanced Normalization Tools (ANTs) and, in particular, the ANTs Cortical Thickness pipeline.^5^ The mean cortical thickness in the whole GM was calculated from the segmentation obtained with the Cortical Thickness pipeline. The segmentation of T2 hyperintensity obtained in step A) was used as a mask for the lesion filling of 3D T1 images. The lesion filling was operated with the lesion filling routine included in Lesion Segmentation Tool.^6^ The Multi-Atlas Label Fusion (MALF) technique implemented in ANTs^7^ was used to parcellate the sub-cortical GM (Thalamus, Putamen, Caudate and Pallidum). The joint label fusion technique was then used to produce the final parcellation labelling.

**CSF analysis**

**Immunoassay protein analysis**

CSF samples were obtained at least 2 months after the last relapse and within one week of the MRI (ethical approval n° 35315), according to Consensus Guidelines for CSF and Blood Biobanking.^8^ After centrifugation, the supernatant and the cell pellet were stored separately at -80°C until use. The IgG index and presence/absence of oligoclonal bands (OCB) for each MS patient is reported in Table 1. The CSF analysis was optimized and performed by two independent investigators (R.M. and S.R.), blinded with respect to the clinical and MRI features.

The levels of 69 inflammatory mediators (Table1) were assessed using a combination of immune-assay multiplex techniques based on the Luminex technology (40- and 37-Plex, Bio-Plex X200 System equipped with a magnetic workstation, BioRad, Hercules, CA, USA) as previously optimized.^9^ All samples were run in duplicate and a number of the molecules were analysed using different immune-assay platforms in order to verify the reproducibility and consistency of the results. The CSF level of each protein detected during the analysis was normalized to the protein concentration of each CSF sample, determined by the Bradford procedure. When comparing the two groups of 14 NIND and 12 OIND controls (Supplementary Table 1), no differences were found in the presence and levels of the examined molecules. Therefore, the two groups were included as a single control group.

The levels of neurofilament light chain (NF-L) in CSF were measured using the Human NF-light enzyme-linked immunosorbent assays (ELISA) kit (MyBioSource, San Diego, CA, USA) and VICTORTM X3 2030 Multilabel Plate Reader (Perkin Elmer, Walluf, Germany) according to the procedures previously optimized.^7,8^

The levels of CSF sCD14 (Quantikine Human sCD14 Immunoassay, R&D Systems), haptoglobin (Quantikine Human Haptoglobin, R&D Systems), free-hemoglobin (HB) (Abcam ab157707) and fibrinogen total antigen (#MBS135523, MyBiosource) were measured in duplicate by ELISA assays according to the manufacturer’s instructions. Samples were analysed in random order and staff was blinded to the treatment arms. Absorbance was measured at 450 nm on a Model 680 Series microplate readers (Bio-Rad).

**Proteomic analysis**

**a) Samples preparation**

Six CSF samples obtained from 3 MSlow and 3 MShigh patients identified as described above (Supplementary Table 2) were selected and analysed onto three different experiments, each of them comparing two patients, one MSlow and one MShigh. For each experimental match, the same amount of total proteins was analyzed (20-30 μg of total proteins, corresponding to a very low amount of CSF from each patient). Each CSF sample was denatured by three different protocols as described,^10^ and the proteome identified by LC-MS/MS with generation of a list of proteins (dataset) for each experiment was obtained. The dataset of identified proteins contained, as expected, large carrier proteins but also proteins derived from brain cells, like for instance S100B, neuronal-specific enolase and pentraxin, confirming that this non-quantitative proteomic approach, based on a multidenaturation step, was comparable with other more sensitive and quantitative approaches, previously published.^10^ The main edge of applying the multi-denaturation protocol, called TRIDENT, consists of maintaining the whole sample (CSF in this work) without any depletion step matched to an increased ability to unfold protein aggregates making them more accessible to proteolytic cleavage, improving discrimination of hidden proteins.^10^ Further bioinformatics and classification analyses were carried out by taking into account the total number of proteins identified by the three denaturation protocols, for each CSF sample. The validation and quantification of the observed proteomic profiles was achieved by means of immunometric assays.

**b) Mass spectrometry analysis**

CSF samples were collected and stored as previously described.^8^ Total protein concentration was measured by means of a Bradford protein assay (Bio-Rad). For each CSF sample three aliquots were obtained and denatured according to three different protocols, as mentioned above (TRIDENT). Protein identification by mass spectrometry analysis was performed as previously described^10^ with the following few modifications: tandem mass spectra were matched against UNIPROT database and through SEQUEST algorithm incorporated in Proteome Discoverer software (version 1.4, Thermo Fisher).

**c) Bioinformatic analysis**

The 227 proteins identified by proteomic analysis (TRIDENT followed by LC-MS/MS) were analyzed by the Database for Annotation, Visualization and Integrated Discovery (DAVID, v6.8) software (https://david.ncifcrf.gov). DAVID provides a comprehensive set of functional annotation tools for investigators to understand biological meaning behind a large list of proteins. Gene ontology analyses were carried out to achieve the Functional Classification of selected proteins, whose gene names were loaded as query using the Gene ID Conversion Tool (only those terms with a p-value ≤0.000001 were selected for DAVID analysis). Protein-protein interactions were studied using STRING software, version 10.5 (<http://string-db.org>) on the basis of confidence (threshold score 0.4 with no more than 10 interactors).^11^

**References**

1. Magliozzi R, Howell OW, Nicholas R, et al. Inflammatory intrathecal profiles and cortical damage in multiple sclerosis. *Ann Neurol* 2018; 83(4):739-755.
2. Calabrese M, Castellaro M, Bertoldo A, et al. Epilepsy in multiple sclerosis: The role of temporal lobe damage. Mult Scler 2016: 1–10.
3. Geurts JJG, et al. Consensus recommendations for MS cortical lesion scoring using double inversion recovery MRI. *Neurology* 2011; 76: 418–24.
4. Calabrese M, et al. A 3-year magnetic resonance imaging study of cortical lesions in relapse-onset multiple sclerosis. *Ann Neurol* 2010; 67: 376–383.
5. Tustison NJ, et al. Large-scale evaluation of ANTs and FreeSurfer cortical thickness measurements. *Neuroimage* 2014; 1;99:166-79.
6. Roura E, et al. A toolbox for segmenting multiple sclerosis lesions using T1w and FLAIR images. *Mult Scler* 2015; 23: 678. (Roura E, Oliver A, Cabezas M, et al. A toolbox for multiple sclerosis lesion segmentation. Neuroradiology 2015;57(10):1031-43. )
7. Wang H, Suh JW, Das SR, et al. Multi-atlas segmentation with joint label fusion. IEEE Trans. *Pattern Anal Mach Intell* 2013; 35: 611–623.
8. Teunissen CE, Petzold A, Bennett JL et al. A consensus protocol for the standardization of cerebrospinal fluid collection and biobanking. *Neurology* 2009; 73: 1914–1922
9. Farina G, Magliozzi R, Pitteri M, et al. Increased cortical lesion load and intrathecal inflammation is associated with oligoclonal bands in multiple sclerosis patients: A combined CSF and MRI study. *J Neuroinflammation* 2017; 14(1)40.
10. Verdoliva V, Senatore C, Polci ML et al. Differential Denaturation of Serum Proteome Reveals a Significant Amount of Hidden Information in Complex Mixtures of Proteins. *PLoS One* 2013; 8(3):e57104.
11. Szklarczyk D, Morris JH, Cook H et al. The STRING database in 2017: Quality-controlled protein-protein association networks, made broadly accessible. *Nucleic Acids Res* 2017; 45: D362–D368.
